# Analysis of the T Cell Response to Zika Virus and Identification of a Novel CD8^+^ T Cell Epitope in Immunocompetent Mice

**DOI:** 10.1371/journal.ppat.1006184

**Published:** 2017-02-23

**Authors:** Ryan D. Pardy, Maaran M. Rajah, Stephanie A. Condotta, Nathan G. Taylor, Selena M. Sagan, Martin J. Richer

**Affiliations:** Department of Microbiology and Immunology, Microbiome and Disease Tolerance Centre, McGill University, Montreal, Quebec, Canada; University of Rochester Medical Center, UNITED STATES

## Abstract

Zika virus (ZIKV) is an emerging arbovirus of the *Flaviviridae* family. Although ZIKV infection is typically mild and self-limiting in healthy adults, infection has been associated with neurological symptoms such as Guillain-Barré syndrome, and a causal link has been established between fetal microcephaly and ZIKV infection during pregnancy. These risks, and the magnitude of the ongoing ZIKV pandemic, have created an urgent need for the development of animal models to study the immune response to ZIKV infection. Previous animal models have primarily focused on pathogenesis in immunocompromised mice. In this study, we provide a model of ZIKV infection in wild-type immunocompetent C57BL/6 mice, and have provided an analysis of the immune response to infection. We evaluated the activation of several innate immune cell types, and studied the kinetics, phenotype, and functionality of T cell responses to ZIKV infection. Our results demonstrate that ZIKV infection is mild in wild-type immunocompetent C57BL/6 mice, resulting in minimal morbidity. Our data establish that at the peak of the adaptive response, antigen-experienced CD4^+^ T cells polarize to a Th1 phenotype, and antigen-experienced CD8^+^ T cells exhibit an activated effector phenotype, producing both effector cytokines and cytolytic molecules. Furthermore, we have identified a novel ZIKV CD8^+^ T cell epitope in the envelope protein that is recognized by the majority of responding cells. Our model provides an important reference point that will help dissect the impact of polymorphisms in the circulating ZIKV strains on the immune response and ZIKV pathogenesis. In addition, the identification of a ZIKV epitope will allow for the design of tetramers to study epitope-specific T cell responses, and will have important implications for the design and development of ZIKV vaccine strategies.

## Author Summary

Zika virus (ZIKV) is a mosquito-borne human pathogen of the *Flaviviridae* family. Most notably, it is responsible for an ongoing epidemic in the Americas, and has been causally linked to birth defects such as fetal microcephaly. These factors have led to an urgent need for small animal models, which may be used to study ZIKV infection, pathogenesis, and the antiviral immune response. To date, the majority of mouse models developed have used mice that lack a competent immune system. These models are excellent for characterizing infection and the pathogenesis of the virus, but are unable to provide a comprehensive analysis of the immune response to ZIKV infection, which will be useful for the design of effective vaccine strategies. Herein, we demonstrate that ZIKV is able to infect wild-type immunocompetent C57BL/6 mice, resulting in activation of the innate immune response and induction of an antiviral T cell response. We have also identified a novel epitope recognized by CD8^+^ T cells within the ZIKV envelope protein. Our model provides an important reference point regarding the T cell response to ZIKV infection, which will be useful in comparative analyses and will have implications in the design and development of effective vaccines.

## Introduction

Zika virus (ZIKV) is an emerging mosquito-borne pathogen that belongs to the *flavivirus* genus of the *Flaviviridae* family, and is related to other globally relevant human pathogens including Dengue, West Nile and Yellow Fever viruses. ZIKV was first described in 1947 after it was isolated from a febrile sentinel monkey in the Zika forest region of Uganda [1, 2]. Although human infection was reported as early as 1964, the first major ZIKV outbreak did not occur until 2007, when nearly 75% of the population of Yap Island, Federated States of Micronesia became infected in a period of 4 months [3–5]. This was followed by an outbreak in French Polynesia in 2013, which marked the first reports of infection-associated neurological symptoms such as Guillain-Barré syndrome and fetal microcephaly [4, 5]. The current ongoing pandemic in the Americas has already seen millions of people infected, and an alarming increase in cases of fetal microcephaly in babies born to mothers infected during pregnancy [4, 5]. Furthermore, the mounting evidence linking ZIKV infection to birth defects has been deemed sufficient to establish a causal relationship [6]. This includes the attenuation of human neural progenitor cell growth *in vitro*, detection of ZIKV in the blood and tissues of microcephalic fetuses, the detection of ZIKV-specific IgM antibodies in the cerebrospinal fluid of microcephalic infants, and intrauterine growth restriction and microcephaly in fetuses of pregnant SJL mice infected with a Brazilian ZIKV isolate [7–10]. Thus, due to the magnitude of the current pandemic and the increase in fetal microcephaly associated with infection, there is an urgent need to develop experimental models for ZIKV to understand infection, tissue tropism, pathogenesis, and the immune response.

Previous studies establishing mouse models of ZIKV infection have primarily been performed in immunocompromised mice; for example, via intracranial infection of suckling mice or in mice deficient in the interferon (IFN)-α/β receptor (IFNAR) or both IFNAR and the IFN-γ receptor (IFNGR) [2, 11–14]. Although very useful for the characterization of pathogenesis, these mice lack an effective immune response, and as such, have limited potential for analysis of the overall immune response to ZIKV infection and the efficacy of vaccine strategies. Furthermore, in some of these models inoculation with as little as one plaque forming unit (PFU) of ZIKV can be lethal [13], which is not reflective of the natural course of infection in immunocompetent adults, where disease is typically mild and self-limiting [4, 5]. The lack of type I IFN also leads to a severely attenuated T cell response, as type I IFN is required for optimal T cell accumulation [15]. A more recent study has successfully demonstrated fetal abnormalities after infection of pregnant wild-type (WT) SJL mice (but not C57BL/6 mice) with a Brazilian ZIKV isolate [10]. Intravaginal infection of WT C57BL/6 mice with ZIKV early in pregnancy has also been demonstrated to cause fetal growth restriction, as has infection of IFNAR^+/-^ heterozygous fetuses [16, 17]. However, analysis of the immune response to ZIKV infection has not been performed to date using these models [10, 16, 17].

Efforts to evaluate the immune response to ZIKV infection have been hampered by a lack of knowledge of specific ZIKV epitopes, which precludes the analysis of antigen-specific T cells using tetramers or peptide restimulation. However, previous studies have established that total T cell responses can be tracked without *a priori* knowledge of specific epitopes using surrogate markers of activation [18, 19]. These studies have thoroughly demonstrated that antigen encounter, but not inflammatory cytokines or bystander infection, leads to a decrease in cell surface expression of CD8α and an increase in surface expression of the integrin CD11a (CD8α^lo^ CD11a^hi^) on CD8^+^ T cells [19]. This phenotype is strictly dependent on antigen-encounter and is maintained for the lifetime of antigen-experienced CD8^+^ T cells, allowing this population of antigen-specific cells to be tracked at late time-points following infection with a variety of pathogens [19, 20]. Similarly, antigen-experienced CD4^+^ T cells can be tracked using surface expression of CD11a and CD49d (CD11a^+^CD49d^+^) following various infections [18, 21, 22]. Thus, this provides a powerful approach to analyze the global T cell response to bacterial, parasitic or viral infections without knowledge of specific epitopes. As such, this is an ideal strategy for studying the immune response to emerging pathogens, such as ZIKV.

The recent demonstration that the ZIKV NS5 protein can block type I IFN production in human cells but not in mouse cells suggests that the IFN response in WT mice may prevent the establishment of infection [23]. In this study, we sought to determine whether immunocompetent WT C57BL/6 adult mice could be infected with ZIKV, and if so, whether the total CD4^+^ and CD8^+^ T cell responses could be tracked using surrogate markers of activation. We observed that infection of C57BL/6 mice induced an antiviral immune response reminiscent of the immune response observed in a variety of other viral infections [24–26]. Furthermore, we have identified a novel, potentially immunodominant, CD8^+^ T cell epitope in the ZIKV envelope protein. Thus, this will serve as an important point of reference for future studies to analyze the immune response to ZIKV infection across virus isolates (to determine the impact of viral evolution), in other mouse strains (to determine the role of host genetics), and will be useful in the identification of additional viral epitopes and in the design and development of effective ZIKV vaccine strategies.

## Results

### ZIKV-Induced T Cell Responses Can Be Tracked in Immunocompetent Mice Using a Surrogate Marker Approach

Previous studies have established that antigen-experienced T cells induced following infection can be tracked without *a priori* knowledge of their specificity using surrogate markers [18, 19]. This approach has allowed analysis of the immune response to infections with a variety of pathogens [18–22]. To further confirm this approach in our mouse colony, we adoptively transferred a low number of congenically marked Thy1.1/1.2 CD4^+^ T cell receptor (TCR) transgenic cells (SMARTA cells, specific to the lymphocytic choriomeningitis virus (LCMV) glycoprotein (GP)_61-80_ peptide) [27] and congenically marked Thy1.2/1.2 CD8^+^ TCR transgenic cells (P14 cells, specific to GP_33-41_ of LCMV) [28], into the same WT Thy1.1/1.1 C57BL/6 recipient mouse and infected the mice intraperitoneally (i.p.) with 2×10^5^ PFU of LCMV Armstrong (acute viral infection that has been extensively used to study T cell responses) the following day. This approach allows us to track cells of known antigen specificity as well as assess both their surrogate marker expression and that of endogenous T cells. We determined the percentage of CD11a^+^CD49d^+^ CD4^+^ T cells and CD8α^lo^CD11a^hi^ CD8^+^ T cells at baseline (day -1) and at the peak of the T cell response, 8 days post-infection (dpi; S1 Fig). As expected, the TCR transgenic CD4^+^ and CD8^+^ T cell populations expanded sufficiently to be detected by flow cytometry, which indicates they have encountered their respective cognate antigens and have been activated. Further, and as described previously, virtually all of the antigen-experienced TCR transgenic CD4^+^ and CD8^+^ T cells responding to infection were identified using the surrogate marker approach (S1C–S1G Fig) [18, 19]. Importantly, and in concurrence with the literature, this method also allowed for the tracking of endogenous antigen-experienced T cells responding to infection [18–22]. Thus, this approach allows us to track endogenous T cell responses to ZIKV infection.

**Fig 1:**
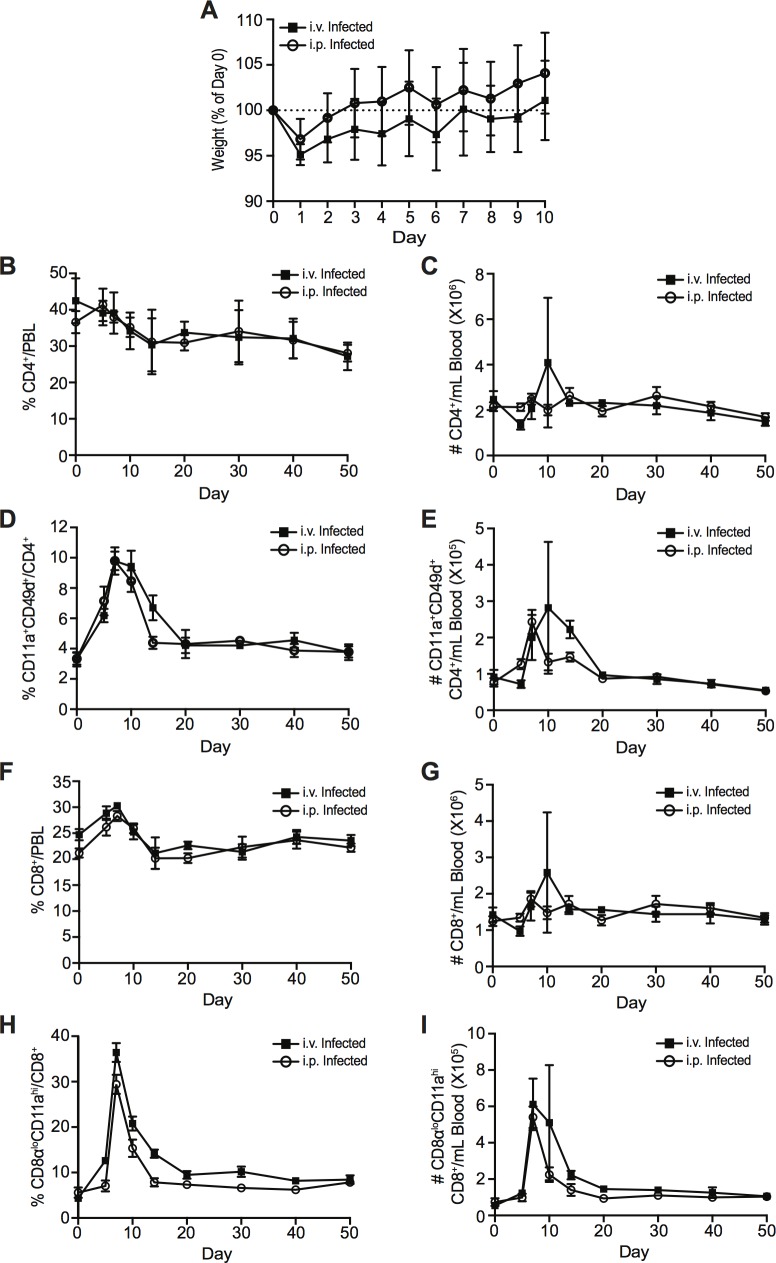
Kinetics of the T cell response following i.v. or i.p. ZIKV infection. **(A)** Weights of i.v.- and i.p.-infected mice presented as a percentage of day 0 weight. Percentage and number of total CD4^+^ T cells **(B-C)**, CD11a^+^CD49d^+^ antigen-experienced CD4^+^ T cells **(D-E)**, total CD8^+^ T cells **(F-G),** and CD8α^lo^CD11a^hi^ antigen-experienced CD8^+^ T cells **(H-I)** at baseline (day 0) and on indicated dpi in the peripheral blood of C57BL/6 mice infected i.p. (open circles) or i.v. (closed squares) with 10^6^ PFU of ZIKV. Error bars represent mean ± SEM. Data has been pooled from two independent experiments, n = 3–4 mice per group per experiment.

WT C57BL/6 adult mice were infected with ZIKV either intravenously (i.v.) or i.p. (Fig 1) with 10^6^ PFU of ZIKV, strain PLCal_ZV, an isolate that is phylogenetically closely related to the French Polynesian strain (Asian lineage), isolated from a Canadian patient who acquired the infection while travelling in Thailand (Genbank accession KF993678) [29]. Although we did not observe any gross behavioural or physical anomalies, ZIKV infection induced mild but detectable morbidity, with mice initially losing weight on day 1 post-infection, before recovering and gradually gaining weight over the following 9 days (Fig 1A). While this represents relatively mild morbidity, the observed weight loss is greater than what is typically observed following LCMV Armstrong infection, which is asymptomatic in mice despite establishing a robust acute infection (S1B Fig). Using the surrogate marker approach, we tracked the induction of ZIKV-specific CD4^+^ and CD8^+^ T cells at various dpi by conducting a longitudinal analysis of the peripheral blood lymphocytes of infected animals. While infection did not dramatically alter the overall proportion or number of total CD4^+^ and CD8^+^ T cells in the blood over time with either route of infection (Fig 1B and 1C and Fig 1F and 1G), we observed the induction of a T cell response that peaked at day 7 post-infection (Fig 1D and 1E and Fig 1H and 1I). Compared to baseline, the proportion of CD11a^+^CD49d^+^ CD4^+^ T cells in i.v.-infected mice increased from approximately 4% (which represents virtual memory cells present in all adult WT mice) [30] to approximately 10% at the peak of the response (7 dpi; Fig 1D). In addition, we observed a greater increase in the proportion of CD8α^lo^CD11a^hi^ CD8^+^ T cells expanding from approximately 6% at baseline (representing the virtual memory population) to approximately 38% on day 7 post-infection (Fig 1H). These were paralleled by a three-fold increase in the number of CD11a^+^CD49d^+^ CD4^+^ T cells, and an eight-fold increase in the number of CD8α^lo^CD11a^hi^ CD8^+^ T cells at the peak of the response (Fig 1E and Fig 1I). The proportions of expanded antigen-experienced T cells underwent a contraction phase, resulting in a pool of presumptive memory CD4^+^ and CD8^+^ T cells, which maintained the CD11a^+^CD49d^+^ or CD8α^lo^CD11a^hi^ phenotype, respectively (Fig 1D and 1E and Fig 1H and 1I). We observed a similar T cell response following i.p. infection, albeit with a slightly reduced magnitude (Fig 1D and 1E and Fig 1H and 1I). Taken together, these results demonstrate that ZIKV infection induces a T cell response in WT immunocompetent mice that can be tracked using the surrogate marker approach, suggesting that this represents a valid model for the analysis of the overall T cell responses to ZIKV infection.

### Kinetics of ZIKV Infection in Adult WT Mice

**Fig 2:**
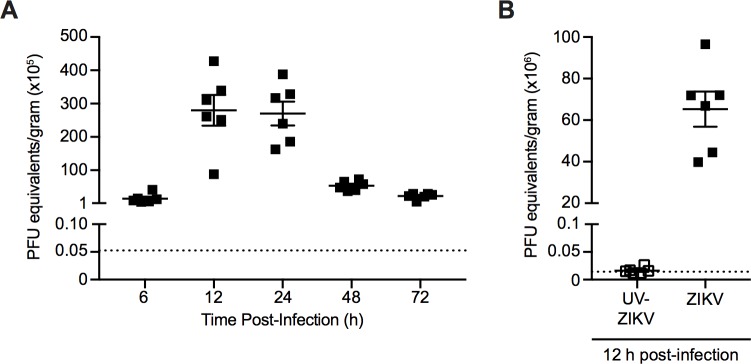
ZIKV infection kinetics. **(A)** Viral RNA was quantified in the spleens of C57BL/6 mice infected i.v. with 10^6^ PFU of ZIKV at 6, 12, 24, 48 and 72 h post-infection using qRT-PCR analysis. **(B)** Viral RNA was quantified in the spleens of C57BL/6 mice infected i.v. with 10^6^ PFU of ZIKV or an equivalent dose of UV-inactivated virus at 12 h post-infection using qRT-PCR analysis. Data are presented as plaque forming unit (PFU) equivalents per gram of tissue after normalization to a standard curve (S2 Fig). The dotted line indicates limit of detection based on the average number of PFU equivalents per gram of tissue from the spleens of mock-infected mice at 48 h post-infection **(A)** or 12 h post-infection **(B)**. Error bars represent mean ± SEM. Data are pooled from two independent experiments, n = 3 mice per group per experiment.

Although the observed T cell response was strongly suggestive of an active viral infection, we sought to determine the kinetics of ZIKV infection by quantifying ZIKV RNA in the spleen at various time points post-infection i.v. with 10^6^ PFU of ZIKV. Spleens were harvested at 6, 12, 24, 48 and 72 hours (h) post-infection, weighed, and total RNA was extracted. Viral burden was assessed using quantitative real-time PCR (qRT-PCR) and the data is expressed as PFU equivalents per gram of tissue (Fig 2A). PFU equivalents were generated by comparing C_t_ values obtained to a standard curve produced using qRT-PCR analysis of ZIKV RNA extracted from titrated viral supernatants previously quantified by plaque assay (S2 Fig).

ZIKV RNA is detectable at 6 h post-infection, with approximately 1.5×10^6^ PFU equivalents/g in the spleen (Fig 2A). ZIKV RNA levels peaked in the spleen at 12 and 24 h post-infection, with an average of approximately 2.8×10^7^ PFU equivalents/g and 2.7×10^7^ PFU equivalents/g, respectively. The level of ZIKV RNA declined steadily thereafter to 2.2×10^6^ PFU equivalents/g at 72 h post-infection. As an additional control, viral RNA was quantified in the spleens of C57BL/6 mice infected i.v. with 10^6^ PFU of ZIKV or an equivalent dose of UV-inactivated virus at 12 h post-infection (peak viral load, Fig 2A) using qRT-PCR (Fig 2B). ZIKV RNA was present at an average of 7×10^7^ PFU equivalents/g in the ZIKV-infected group, and was virtually undetectable in the UV-inactivated group (Fig 2B). This indicates that infection with live virus is required to detect viral RNA in the spleen at the tested time points (Fig 2). Thus, our data demonstrates that ZIKV can infect WT immunocompetent mice resulting in the accumulation of viral RNA in the spleen that peaks at 12–24 h post-infection.

### ZIKV Infection Induces Dendritic Cell and Natural Killer Cell Activation

**Fig 3:**
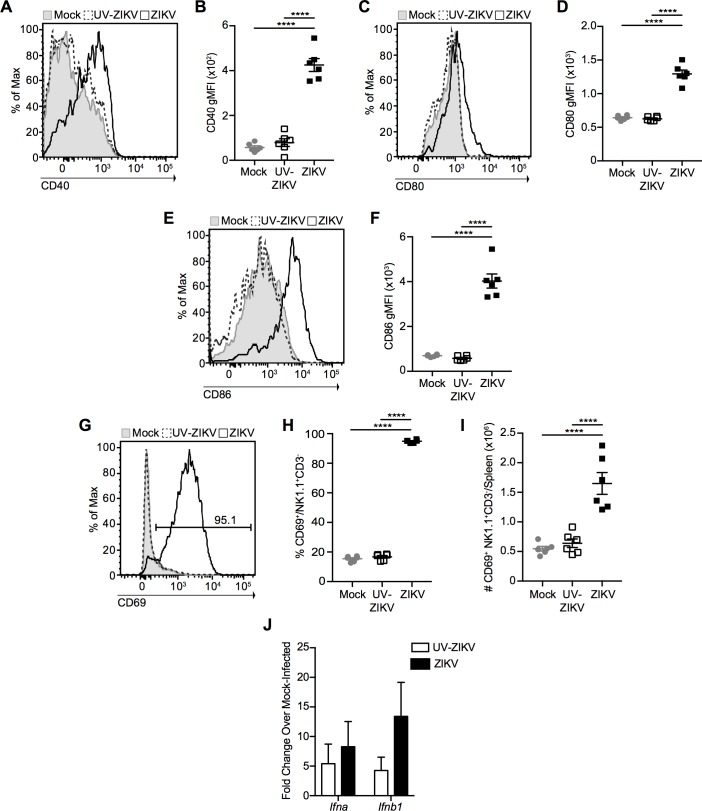
ZIKV induces innate immune cell activation. Representative histograms and geometric mean fluorescence intensity (gMFI) of CD40 **(A**-**B)**, CD80 **(C**-**D)**, and CD86 **(E-F)** on splenic dendritic cells (DCs) at 2 dpi with 10^6^ PFU of ZIKV i.v. (open histograms with solid line), an equivalent dose of UV-inactivated ZIKV (open histograms with dashed line) or mock infection (shaded histograms). Histograms represent DCs gated as CD3^-^ CD19^-^ NK1.1^-^ MHC-II^+^ CD11c^+^ cells. Representative histogram **(G)**, percentage **(H)**, and number **(I)** of CD69^+^ NK1.1^+^CD3^-^ natural killer cells from the spleens of ZIKV- (open histogram with solid line), UV-inactivated ZIKV- (open histogram with dashed line) and mock-infected (shaded histogram) mice 2 dpi. Number on histogram indicates percentage of CD69^+^ cells from ZIKV-infected mouse. **(J)** Mice were infected with 10^6^ PFU of ZIKV or an equivalent dose of UV-inactivated virus, spleens were harvested 12 h post-infection and total RNA was extracted. IFN-α and IFN-β mRNA expression was assessed by qRT-PCR, normalized to TATA-binding protein mRNA expression, and expressed as fold change over mRNA expression in mock-infected mice. Error bars represent mean ± SEM. Data in (**A**-**I**) are pooled from two independent experiments, n = 3 mice per group per experiment. Data in (**J**) are pooled from three independent experiments, n = 3 mice per group per experiment. Data in **(B**, **D**, **F**, **H** and **I)** were analyzed by one-way ANOVA with Tukey’s post-test of multiple comparisons. ****p<0.0001.

As our data demonstrated that ZIKV was able to infect WT mice, we next sought to analyze the innate immune response to infection by measuring dendritic cell (DC) and natural killer (NK) cell activation. C57BL/6 mice were infected i.v. with 10^6^ PFU of ZIKV, an equivalent dose of UV-inactivated ZIKV, or mock-infected, and spleens were harvested at 2 dpi. ZIKV induced a significant increase in the expression of the co-stimulatory molecules CD40, CD80, and CD86 compared to mock- or UV-inactivated virus-infected mice (Fig 3A–3F), indicating that DC activation is dependent on infection with live virus (the gating strategy used to identify DCs is indicated in S3A–S3D Fig). Furthermore, NK cells in the spleens of ZIKV-infected mice also presented with an activated phenotype as measured by a significant increase in the percentage and number of NK cells expressing the activation marker CD69 (Fig 3G–3I). Finally, we assessed early total NK1.1^-^CD3^+^ T cell activation using expression of the early activation marker CD69 at 2 dpi. In ZIKV-infected mice, but not those infected with UV-inactivated virus or mock-infected, we observed a significant increase in the percentage and number of CD69^+^ total T cells, suggestive of early T cell activation (S3E–S3G Fig). However, it is important to note that this could be a result of antigen or cytokine encounter at this early time-point. Thus, ZIKV infection of WT mice induces innate immune cell activation that is dependent upon infection with live virus.

The importance of the type I IFN response in ZIKV immunity has been demonstrated clearly by the severe morbidity and mortality of mice lacking IFNAR or both IFNAR and IFNGR in previous models of ZIKV infection [11–14]. To assess type I IFN production in response to ZIKV infection, we infected mice i.v. with 10^6^ PFU of ZIKV or an equivalent dose of UV-inactivated virus and harvested spleens at 12 h post-infection. Total RNA was extracted, reverse-transcribed, and analyzed for IFN-α and IFN-β gene expression using qRT-PCR. We observed induction of both IFN-α and IFN-β in both groups compared to mock-infected mice, suggesting that, as opposed to DC and NK cell activation, infection with live virus is not a strict requirement for the induction of a type I IFN response (Fig 3J). Thus, ZIKV infection induces a rapid type I IFN response that likely plays a role in limiting viral replication in this model.

### CD4^+^ T Cells Polarize to a Th1 Phenotype in Response to ZIKV Infection

**Fig 4:**
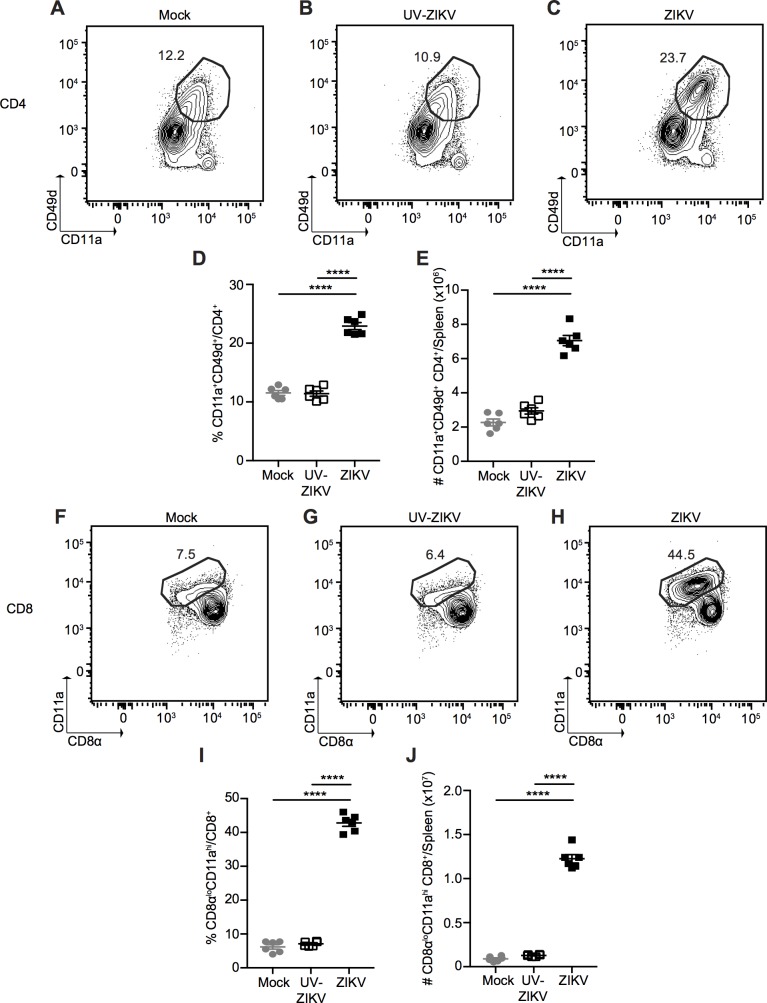
ZIKV induces CD4^+^ and CD8^+^ T cell activation at the peak of the T cell response. Representative plots of CD11a^+^CD49d^+^ antigen-experienced CD4^+^ T cells from spleens of mock- **(A)**, UV-inactivated ZIKV- **(B)** and ZIKV-infected **(C)** mice 7 dpi. Percentage **(D)** and number **(E)** of CD11a^+^CD49d^+^ antigen-experienced CD4^+^ T cells from spleens of mock-, UV-inactivated ZIKV- and ZIKV-infected mice 7 dpi. Representative plots of CD8α^lo^CD11a^hi^ antigen-experienced CD8^+^ T cells from the spleens of mock- **(F)**, UV-inactivated ZIKV- **(G)** and ZIKV-infected **(H)** mice 7 dpi. Percentage **(I)** and number **(J)** of CD8α^lo^CD11a^hi^ antigen-experienced CD8^+^ T cells from the spleens of mock-, UV-inactivated ZIKV- and ZIKV-infected mice 7 dpi. Error bars represent mean ± SEM. Data are pooled from two independent experiments, n = 3 mice per group per experiment. Data in **(D**, **E**, **I** and **J)** were analyzed by one-way ANOVA with Tukey’s post-test of multiple comparisons. ****p<0.0001.

To further characterize the nature of the T cell response induced following ZIKV infection, we infected mice i.v. with 10^6^ PFU of ZIKV, an equivalent dose of UV-inactivated virus or mock-infected, and harvested the spleens at 7 dpi (peak of the T cell response, Fig 1). Similar to what we observed in the peripheral blood, ZIKV infection induced a CD4^+^ T cell response in the spleen with a significant increase in both the frequency and number of antigen-experienced (CD11a^+^CD49d^+^) CD4^+^ T cells (Fig 4A–4E), compared to UV-inactivated virus- and mock-infected mice (27% versus 12% and 11%, respectively).

Additionally, we examined the CD4^+^ T cell response to varying doses of ZIKV to determine whether the observed immune response is dose-dependent (S4 Fig). Mice were infected i.v. with 10^4^, 10^5^ or 10^6^ PFU of ZIKV or mock-infected, and CD4^+^ T cell activation was assessed 7 dpi. ZIKV-infected mice exhibited significantly higher CD4^+^ T cell activation than mock-infected mice at all doses tested. Although there were no significant differences in the proportions of antigen-experienced CD4^+^ T cells in response to the various doses of ZIKV, we observed a significant reduction in the number of CD11a^+^CD49d^+^ CD4^+^ T cells in the mice infected with 10^5^ PFU of ZIKV compared to those infected with 10^6^ PFU (S4A–S4F Fig). Thus, the magnitude of the CD4^+^ T cell response to ZIKV is dependent on the dose of inoculation. This further validates our use of 10^6^ PFU, which ensured infection and induced a more robust immune response than the lower doses tested.

In the presence of different inflammatory cytokines, CD4^+^ T cells can be polarized into one of several T helper (Th) subtypes. Briefly, in the presence of interleukin (IL)-12 and IFN-γ, which are typically produced in response to viral infection, effector CD4^+^ T cells polarize to the Th1 subset, characterized by production of IFN-γ, tumour necrosis factor (TNF)-α and IL-2, and expression of the T-box transcription factor, T-bet [31]. In contrast, IL-4 is critical for the development of Th2 cells, which are characterized by the production of IL-5, among other cytokines. Finally, both IL-23 and tumour growth factor (TGF)-β are important for the development and maintenance of to Th17 cells, which produce mainly IL-17 [31].

**Fig 5:**
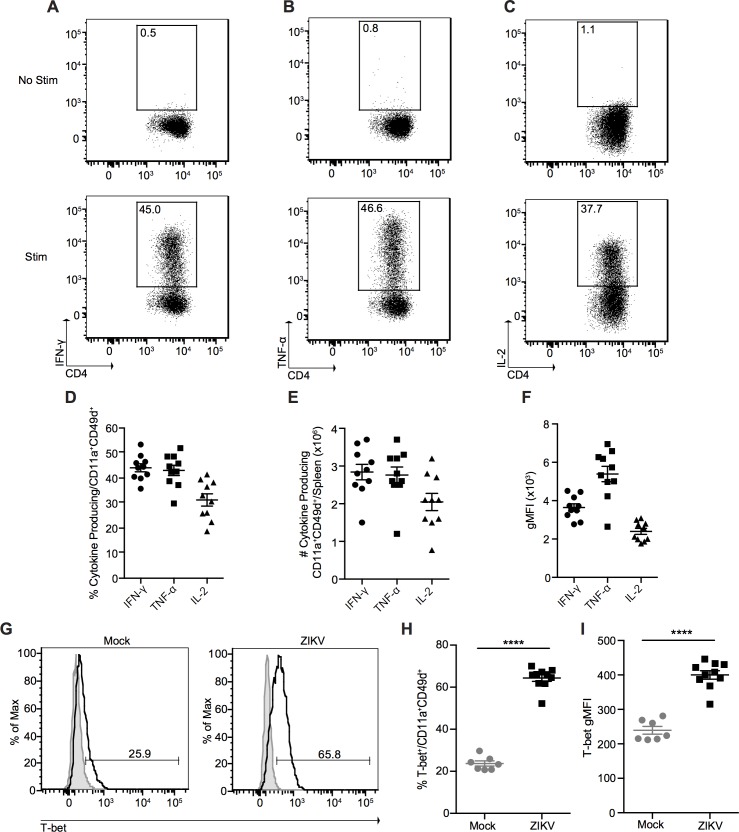
ZIKV infection induces a Th1 CD4^+^ T cell response. Representative plots of IFN-γ **(A)**, TNF-α **(B)**, and IL-2 **(C)** production from splenic CD11a^+^CD49d^+^ CD4^+^ T cells from ZIKV-infected mice 7 dpi. Total splenocytes were incubated in media alone in the presence of Brefeldin A (top) or stimulated with PMA and ionomycin in the presence of Brefeldin A for 3 h at 37°C (bottom). Percentage **(D)**, number **(E)**, and gMFI **(F)** of cytokine-producing CD11a^+^CD49d^+^ CD4^+^ T cells from the spleens of ZIKV-infected mice 7 dpi. **(G)** Representative histograms of T-bet expression in splenic CD11a^+^CD49d^+^ CD4^+^ T cells from mock- and ZIKV-infected mice 7 dpi. Shaded histograms represent isotype control. Percentage **(H)** and gMFI **(I)** of T-bet expression in splenic CD11a^+^CD49d^+^ CD4^+^ T cells from mock- and ZIKV-infected mice 7 dpi. Error bars represent mean ± SEM. Data are pooled from two independent experiments, n = 3–5 mice per group per experiment. Data in **(H** and **I)** were analyzed with a two-tailed, unpaired Student’s t test. ****p<0.0001.

To determine the differentiation status of the effector CD4^+^ T cells induced by ZIKV infection, we stimulated total splenocytes from ZIKV-infected mice with phorbol myristate acetate (PMA) and ionomycin in the presence of Brefeldin A for 3 h and analyzed cytokine production with intracellular cytokine staining via flow cytometry (Fig 5). We observed that antigen-experienced CD4^+^ T cells exhibited a typical Th1 cytokine profile characterized by the production of IFN-γ, TNF-α, and IL-2 in response to PMA and ionomycin stimulation (but not in the absence of stimulation) (Fig 5A–5F). As an additional control, we also stimulated total splenocytes with plate-bound anti-CD3 antibody for 5 h in the presence of Brefeldin A. Since naïve T cells are dependent on CD28 co-stimulation for cytokine production, only effector T cells are able to respond to CD3 cross-linking [32]. As expected, IFN-γ production was only detectable from CD11a^+^CD49d^+^ CD4^+^ T cells, and not naïve CD11a^-^CD49d^-^ CD4^+^ T cells (S5A–S5C Fig). In addition, a significantly higher proportion of CD11a^+^CD49d^+^ CD4^+^ T cells expressed the Th1 transcription factor T-bet, and significantly more T-bet on a per-cell basis when compared with mock-infected mice (Fig 5G–5I).

The Th1 cells induced by ZIKV infection also exhibited a high degree of poly-functionality (multiple cytokine production), as over 65% of responding CD11a^+^CD49d^+^ CD4^+^ T cells were IFN-γ^+^TNF-α^+^ double-producers, and over 60% were IFN-γ^+^TNF-α^+^IL-2^+^ triple-producers (S6A–S6D Fig). We also observed no production of either the Th2 cytokine IL-5 or the Th17 cytokine IL-17 (S7A–S7E Fig), further confirming that ZIKV infection induces a Th1-polarized response.

Although the production of Th1, but not Th2, cytokines has previously been described in response to LCMV infection [25], we sought to characterize the cytokine profile of antigen-experienced CD4^+^ T cells in LCMV-infected mice in order to compare what was observed in ZIKV-infected mice to a known model pathogen. As expected, and in concordance with the literature [25], antigen-experienced CD4^+^ T cells from LCMV-infected mice produced only IFN-γ, TNF-α and IL-2, but not the Th2 cytokine IL-5 or Th17 cytokine IL-17, in response to PMA and ionomycin stimulation (but not in the absence of stimulation) (S8 Fig). Taken together, these results establish that ZIKV infection induces a prototypical Th1 CD4^+^ T cell response, without inducing substantial Th2 or Th17 responses.

In addition to inducing effector CD4^+^ T cells, viral infection can have an impact on regulatory T cells (Tregs), a T cell subset that plays a key role in the maintenance of immune homeostasis [33]. ZIKV infection led to a significant decrease in the percentage of splenic FoxP3^+^ Tregs, although this was accompanied by a significant increase in the total number of Tregs present in the spleen (S7F–S7I Fig). Thus, ZIKV infection may have an impact on Treg numbers and it will be of interest to determine what role, if any, this plays in the natural course of ZIKV infection.

### ZIKV Infection Induces a Robust Effector CD8^+^ T Cell Response

To assess the function and phenotype of the ZIKV-specific CD8^+^ T cells at the peak of the response, we first assessed the proportion of antigen-experienced (CD8α^lo^CD11a^hi^) CD8^+^ T cells in the spleen at 7 dpi using the surrogate marker approach. Similar to what we observed in the peripheral blood, ZIKV infection induces a robust CD8^+^ T cell response with a significant increase in the proportion of antigen-experienced CD8^+^ T cells compared to UV-inactivated virus or mock-infection (41% versus 7% and 8% respectively, Fig 4F–4I), resulting in an approximately eight-fold increase in total number of CD8α^lo^CD11a^hi^ CD8^+^ T cells (Fig 4J). This response was also dose-dependent, as infecting with 10^4^ PFU of ZIKV induced a lower number of CD8α^lo^CD11a^hi^ CD8^+^ T cells than infection with 10^5^ or 10^6^ PFU, despite no difference in the proportion of antigen-experienced CD8^+^ T cells across doses (S4G–S4L Fig).

**Fig 6:**
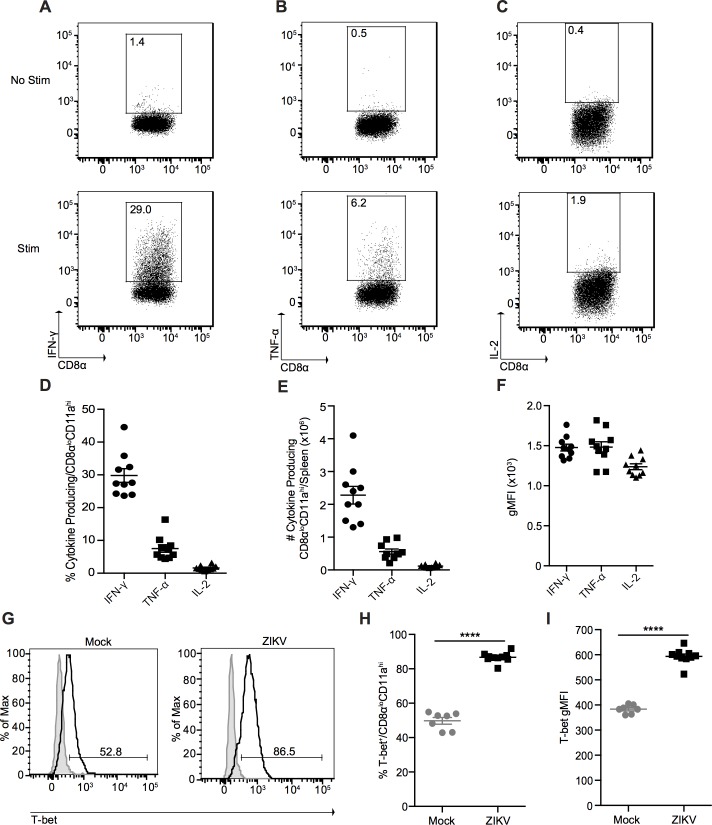
ZIKV infection induces a prototypical effector CD8^+^ T cell response in immunocompetent mice. Representative plots of IFN-γ **(A)**, TNF-α **(B)**, and IL-2 **(C)** production from splenic CD8α^lo^CD11a^hi^ CD8^+^ T cells from ZIKV-infected mice 7 dpi. Total splenocytes were incubated in media alone in the presence of Brefeldin A (top) or stimulated with PMA and ionomycin in the presence of Brefeldin A for 3 h at 37°C (bottom). Percentage **(D)**, number **(E)**, and gMFI **(F)** of cytokine-producing CD8α^lo^CD11a^hi^ CD8^+^ T cells from the spleens of ZIKV-infected mice 7 dpi. **(G)** Representative histograms of T-bet expression in splenic CD8α^lo^CD11a^hi^ CD8^+^ T cells from mock- and ZIKV-infected mice 7 dpi. Shaded histograms represent isotype control. Percentage **(H)** and gMFI **(I)** of T-bet expression in splenic CD8α^lo^CD11a^hi^ CD8^+^ T cells from mock- and ZIKV-infected mice 7 dpi. Error bars represent mean ± SEM. Data are pooled from two independent experiments, n = 3–5 mice per group per experiment. Data in **(H** and **I)** were analyzed with a two-tailed, unpaired Student’s t test. ****p<0.0001.

Next, we determined the capacity of the CD8α^lo^CD11a^hi^ CD8^+^ T cells to produce key effector cytokines such as IFN-γ and TNF-α, as well as IL-2. Following PMA and ionomycin stimulation, antigen-experienced CD8^+^ T cells responded by producing IFN-γ, with nearly 20% of cells maintaining some level of poly-functionality, producing both IFN-γ and TNF-α (Fig 6A, 6B and 6D–6F and S6E and S6F Fig). Furthermore, plate-bound anti-CD3 stimulation led to cytokine production only from the antigen experienced CD8α^lo^CD11a^hi^ CD8^+^ T cells, and not from naïve CD8α^hi^CD11a^lo^ CD8^+^ T cells (S5D–S5F Fig). As expected for effector CD8^+^ T cells, we did not observe any IL-2 production from the antigen-experienced CD8^+^ T cells (Fig 6C–6F). These results are highly reminiscent to what has been described in the immune response to LCMV infection, which induces production of IFN-γ and TNF-α, and minimal IL-2 production [24]. We also confirmed that this cytokine profile is indeed observed in CD8^+^ T cells exhibiting the antigen-experienced phenotype in LCMV infection (S9 Fig). Finally, the majority of antigen-experienced CD8^+^ T cells expressed the transcription factor T-bet, which is critical for effector CD8^+^ T cell function (Fig 6G–6I) [34].

**Fig 7:**
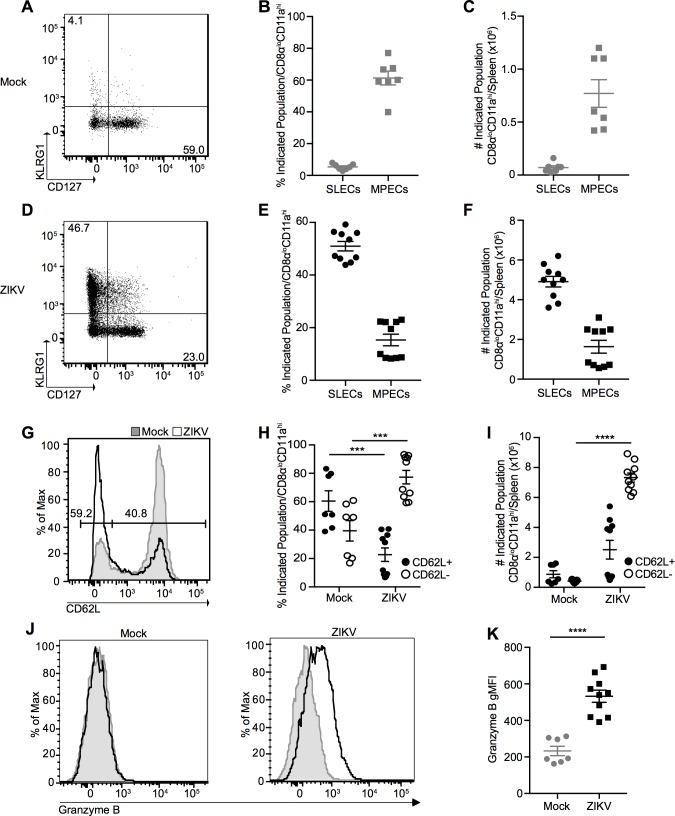
Antigen-experienced CD8^+^ T cells present with an activated phenotype at the peak of the T cell response. **(A)** Representative plot of CD127 and KLRG1 expression on CD8α^lo^CD11a^hi^ CD8^**+**^ T cells from spleens of mock-infected mice 7 dpi. Percent **(B)** and number **(C)** of CD127^lo^KLRG1^hi^ short-lived effector cells (SLECs) and CD127^hi^KLRG1^lo^ memory precursor effector cells (MPECs) on CD8α^lo^CD11a^hi^ CD8^**+**^ T cells from mock-infected mice. **(D)** Representative plot of CD127 and KLRG1 expression on CD8α^lo^CD11a^hi^ CD8^**+**^ T cells from spleens of ZIKV-infected mice 7 dpi. Percent **(E)** and number **(F)** of SLECs and MPECs on CD8α^lo^CD11a^hi^ CD8^**+**^ T cells from ZIKV-infected mice. Representative histogram **(G)**, percentage **(H)**, and number **(I)** of CD62L^+^ and CD62L^-^ CD8α^lo^CD11a^hi^ CD8^**+**^ T cells from spleens of mock- (shaded histogram) and ZIKV-infected (open histogram) mice 7 dpi. Numbers on histogram indicate percentage of CD62L^-^ (left gate) and CD62L^+^ (right gate) cells from ZIKV-infected sample. **(J)** Representative histograms of granzyme B expression in CD8α^lo^CD11a^hi^ CD8^**+**^ T cells from spleens of mock- (left) and ZIKV-infected (right) mice 7 dpi. Shaded histograms represent isotype control. **(K)** gMFI of granzyme B expression. Error bars represent mean ± SEM. Data are pooled from two independent experiments, n = 3–5 mice per group per experiment. Data in **(H**, **I** and **K)** were analyzed with a two-tailed, unpaired Student’s t test. ***p<0.0005; ****p<0.0001.

Viral infection typically induces CD8^+^ T cell populations that can be broadly classified into either short-lived effector cells (SLECs) that are critical for the acute immune response or memory precursor effector cells (MPECs) that preferentially survive contraction and go on to seed the memory CD8^+^ T cell pool [34]. As expected for the peak of the immune response where SLECs are critical for protective responses, the majority of cells polarized to a SLEC phenotype following ZIKV infection, expressing high levels of killer cell lectin-like receptor G1 (KLRG1) and down-regulating CD127 (IL-7 Receptor α chain); while only approximately 25% of cells maintained an MPEC phenotype (Fig 7D–7F). As demonstrated previously [34], the balance of these two cell populations is dynamic and changes over time post-infection, with the majority of CD8α^lo^CD11a^hi^ CD8^**+**^ T cells expressing an MPEC phenotype, and less than 25% retaining the SLEC phenotype at >100 dpi (S10 Fig). Furthermore, antigen-experienced CD8^+^ T cells significantly down-regulate CD62L (L-selectin) (Fig 7G–7I), a lymph node homing marker that is characteristic of central memory CD8^+^ T cells [35], and ZIKV infection results in significantly more granzyme B (an important cytolytic molecule) expression on a per-cell basis than mock-infected mice (Fig 7J and 7K) at the peak of the T cell response. In contrast, the small population of CD8α^lo^CD11a^hi^ CD8^+^ T cells from mock-infected mice retain a memory-like phenotype (likely virtual memory), expressing little KLRG1 and high levels of CD127 and CD62L, demonstrating that these cells are not activated during mock-infection (Fig 7A–7C and 7G–7I). Importantly, not all antigen-experienced effector cells can be classified as CD62L^-^ or granzyme B^+^ further emphasizing the power of the surrogate marker approach able to track all antigen-experienced cells regardless of their activation phenotype. In addition, the proportion of SLEC/MPECs at the peak of infection is reminiscent of the profile reported for CD8^+^ T cells responding to LCMV infection [34]. Taken together, our data indicates that ZIKV infection is able to induce a prototypical antiviral CD8^+^ T cell response in WT immunocompetent mice characterized by the majority of the antigen-specific CD8^+^ T cells developing critical effector functions.

### Antigen-Experienced CD8^+^ T Cells Recognize an Epitope in the ZIKV Envelope Protein

In order to provide further validation of the surrogate marker approach in the analysis of the immune response to ZIKV infection, as well as to potentially identify ZIKV-specific CD8^+^ T cell epitopes, we used the Immune Epitope Database (IEDB) analysis resource consensus tool to predict CD8^+^ T cell epitopes within the ZIKV polyprotein [36]. This tool identifies peptides that are likely to bind the H-2-Db allele of class I major histocompatibility complex (MHC-I) in mice. From this list, we chose to further analyze the top six predicted peptides, including Env_294-302_, NS2A_1272-1280_, NS2A-NS2B_1350-1358_, NS4B_2329-2337_, NS4B_2371-2379_, and NS5_3066-3074_ (numbered for their position in the ZIKV MR766 polyprotein, Genbank accession LC002520).

**Fig 8:**
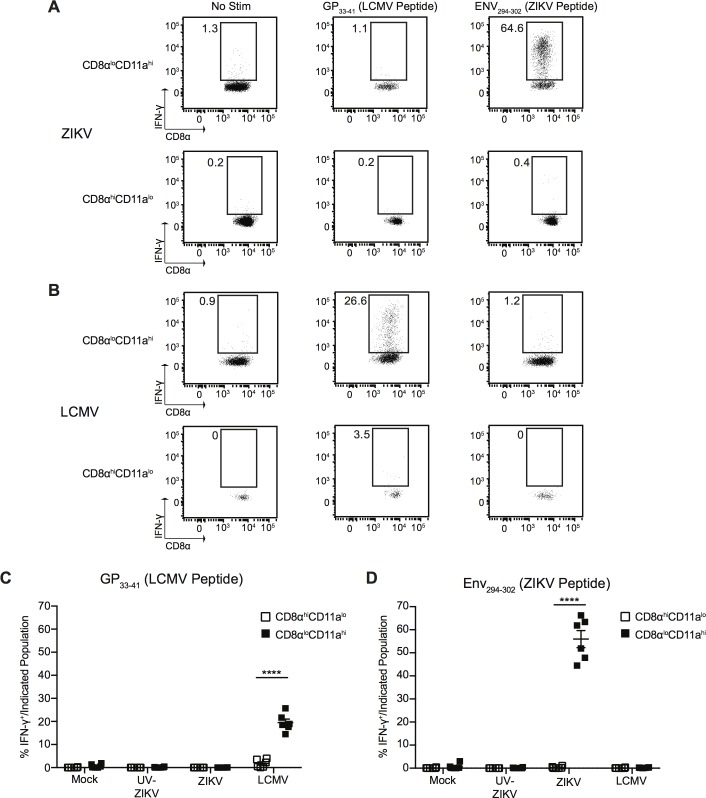
CD8^+^ T cells respond to an epitope within the ZIKV Envelope protein. **(A)** Representative plots of IFN-γ production from antigen-experienced CD8α^lo^CD11a^hi^ CD8^+^ T cells (top row) and naïve CD8α^hi^CD11a^lo^ CD8^+^ T cells (bottom row) from the spleens of ZIKV-infected mice 7 dpi. Total splenocytes were incubated for 5.5 h at 37°C with media alone or 200nM of the indicated peptide in the presence of Brefeldin A. **(B)** Representative plots of IFN-γ production from antigen-experienced CD8α^lo^CD11a^hi^ CD8^+^ T cells (top row) and naïve CD8α^hi^CD11a^lo^ CD8^+^ T cells (bottom row) from the spleens LCMV-infected mice 7 dpi. Total splenocytes were incubated for 5.5 h at 37°C with media alone or 200nM of the indicated peptide in the presence of Brefeldin A. **(C-D)** Percentage of IFN-γ^+^ antigen-experienced CD8α^lo^CD11a^hi^ or naïve CD8α^hi^CD11a^lo^ CD8^+^ T cells from mock-, UV-inactivated ZIKV-, ZIKV- or LCMV-infected mice 7 dpi after restimulation with media alone or 200nM of GP_33-41_ (LCMV peptide) **(C)** or Env_294-302_ (ZIKV peptide) **(D)** for 5.5 h at 37°C in the presence of Brefeldin A. Error bars represent mean ± SEM. Data are pooled from two independent experiments, n = 3 mice per group per experiment. Data in **(C** and **D)** were analyzed with a two-tailed, paired Student’s t test. ****p<0.0001.

Next, we infected mice with 10^6^ PFU of ZIKV, an equivalent dose of UV-inactivated virus, mock infection, or 2x10^5^ PFU of LCMV Armstrong. At the peak of the T cell response to ZIKV (7 dpi) we sacrificed the mice, harvested spleens, and restimulated total splenocytes for 5.5 h in the presence of Brefeldin A with media alone, GP_33-41_ (an immunodominant epitope of LCMV), or with one of the ZIKV-specific peptides. After incubation, the capacity of each peptide to stimulate IFN-γ production from the antigen-experienced (CD8α^lo^CD11a^hi^) and naïve (CD8α^hi^CD11a^lo^) CD8^+^ T cells was assessed by intracellular staining using flow cytometry (Fig 8, S11 Fig and S12 Fig). In the LCMV-infected group, IFN-γ production from antigen-experienced (CD8α^lo^CD11a^hi^) CD8^+^ T cells was only observed after restimulation with GP_33-41_, and not with any of the ZIKV peptides (Fig 8B–8D and S12B–S12G Fig). In the ZIKV-infected group, we observed a strong induction of IFN-γ production from antigen-experienced (CD8α^lo^CD11a^hi^) CD8^+^ T cells only when stimulated with the Env_294-302_ peptide, with an average of approximately 56% of cells producing IFN-γ (Fig 8A, 8C and 8D and S12A, S12C–S12G Fig). Neither the mock-infected group, nor the group infected with UV-inactivated virus, responded to stimulation with the Env_294-302_ peptide (Fig 8C and 8D and S11 Fig). Importantly, we observed no production of IFN-γ from naïve CD8α^hi^CD11a^lo^ CD8^+^ T cells in response to any peptides, in any of the infection groups (Fig 8, S11 Fig and S12 Fig). This indicates that Env_294-302_ is a *bona fide* ZIKV epitope recognized by CD8^+^ T cells, and suggests that it is the immunodominant epitope recognized by CD8^+^ T cells in this model. This data also further validates the use of the surrogate marker approach for tracking the total T cell response to ZIKV infection and directly identifies a novel ZIKV epitope targeted by CD8^+^ T cells.

## Discussion

A rapid and effective global response will be required for the development of vaccines and antiviral therapies to combat the ongoing ZIKV epidemic in the Americas where viral pathogenesis appears to be increasing in severity. Paramount to this goal is the establishment of suitable small animal models to study ZIKV pathogenesis and the immune response to infection. Despite the development of several small animal models to study ZIKV infection and pathogenesis, most of these models lack critical innate immune components and succumb rapidly to ZIKV infection, precluding an analysis of the immune response to infection [11–14, 16, 17]. As such, the model developed and data presented herein represent an important reference point for the analysis of the immune response to ZIKV infection in adult, immunocompetent mice, and has uncovered a novel epitope of ZIKV recognized by CD8^+^ T cells.

The recent demonstration that the ZIKV NS5 protein is capable of interrupting type I IFN production in human, but not mouse cells has led to the suggestion that ZIKV is unable to cause disease in mice [23]. However, our data demonstrates that ZIKV establishes a limited, but detectable, infection in immunocompetent WT C57BL/6 adult mice resulting in minimal morbidity. This is supported by data from other studies where ZIKV RNA was detectable in the spleen, albeit at low levels, following infection of either 129Ev/Sv or C57BL/6 mice, both immunocompetent mouse strains [11, 13]. More importantly, we show that ZIKV infection of adult WT C57BL/6 mice is sufficient to induce a T cell response, which peaks at day 7 before contracting to potentially form a pool of memory T cells. This course of infection in immunocompetent mice is potentially more representative of typical ZIKV pathogenesis in immunocompetent adult humans, which is typically mild and self-limiting [4, 5]. Furthermore, since type I IFN signalling is required for initiation of optimal T cell responses [15], immunocompetent mouse models will be extremely valuable for vaccine strategies aimed at inducing T cell responses, particularly as this model has enabled us to identify a novel ZIKV epitope that could be used in vaccination approaches.

Our model of ZIKV infection combined with the surrogate marker approach provides a valuable reference point that is required to begin interrogating whether pathogenic isolates impact the host immune response to establish a more severe infection. Additionally, the current approach will allow us to compare the immune response induced by infection in a variety of mouse strains (where epitopes are unlikely to be conserved) and potentially link the immune response back to pathogenesis. The recent demonstration that infection of pregnant SJL, but not C57BL/6, mice with a Brazilian ZIKV isolate results in intrauterine growth restriction and microcephaly raises several questions regarding ZIKV evolution and host genetics that can be addressed in our model [10]. Importantly, the approaches developed by Rai *et al*. and McDermott *et al*. allow us to measure and compare the immune response in strains with different genetic backgrounds [18, 19]. Thus, the combination of this approach, which we have now thoroughly validated for use in ZIKV infection models, and the use of reverse genetics systems to manipulate viral sequences will allow us to answer a variety of important questions in the future [37, 38].

The identification of a specific, potentially immunodominant, ZIKV epitope in the envelope protein provides an important advancement for ZIKV immunology. Interestingly, this epitope is conserved across 99% (103/104) of all full-length ZIKV polyprotein sequences available on Genbank to date, making it a powerful tool for future studies. Knowledge of the epitopes to which the immune system responds will allow for the design of ZIKV vaccine candidates aimed at inducing a potent T cell response, and will lead to the establishment of tetramers to identify and track Env_294-302_-specific CD8^+^ T cells. It will also be of interest to determine whether this epitope is immunogenic in human samples from the ongoing epidemic.

In conclusion, our findings demonstrate that ZIKV infection of immunocompetent adult C57BL/6 mice is a self-limiting infection inducing mild, but detectable, morbidity. This induces innate immune activation, leading to a Th1-polarized CD4^+^ T cell response and an effector CD8^+^ T cell response. By tracking the total T cell response using the surrogate marker approach, we have identified a novel CD8^+^ T cell epitope in the ZIKV envelope protein that will have important implications for ZIKV immunology and the design and testing of ZIKV vaccine candidates.

## Methods

### Cells

Vero cells (African Green monkey kidney epithelial cells, kindly provided by Steven Varga, University of Iowa) were cultured in DMEM supplemented with 10% heat-inactivated fetal bovine serum (FBS), 1% L-glutamine and 1% non-essential amino acids at 37°C and 5% CO_2_.

### Viruses

Low passage (p.4) infectious ZIKV (PLCal_ZV, Genbank accession KF99378) derived from a ZIKV-infected traveller returning to Canada was kindly provided by the National Microbiology Laboratory (Winnipeg, Canada) [29]. ZIKV stocks were propagated in Vero cells after infecting at a multiplicity of infection (MOI) of 0.5. Supernatants from both mock-infected as well as ZIKV-infected cells were harvested after 72 h post-infection, filtered, and viral stocks were titrated by plaque assay on Vero cells. Briefly, Vero cells were seeded at a density of 1.5×10^6^ cells per 60 mm dish. Twenty-four hours later, viral stocks were serially diluted (10-fold) in EMEM and 2 mL of each dilution was used for infection. At 2 h post-infection, cells were overlaid with EMEM (Wisent), 1.2% Carboxymethyl cellulose (Sigma-Aldrich), and 2% heat-inactivated FBS for 4 days prior to fixation with 5% formaldehyde and 0.1% crystal violet stain. ZIKV was UV-inactivated by transferring 1 mL of ZIKV into one well of a 6-well plate and exposing it to 3 Joules/cm^2^ of UV irradiation in a UVC 500 Crosslinker (Hoefer). Dose of UV irradiation was chosen based on previous work in related flaviviruses [39]. LCMV Armstrong was kindly provided by John Harty (University of Iowa) and propagated as described [40].

### Mouse Experiments

C57BL/6 mice (WT) were purchased from Charles River laboratories or bred at McGill University. Infected mice were housed at the appropriate biosafety level and used at 6–9 weeks of age. ZIKV (p.7) was injected i.v. or i.p. at 1×10^4^, 1×10^5^, or 1×10^6^ PFU in a final volume of 200 μL of Phosphate Buffered Saline (PBS). Mice infected with UV-inactivated ZIKV received a dose of inactivated virus equivalent to 1×10^6^ PFU diluted in PBS to a final volume of 200 μL. Mock-infected mice received an identical volume of mock-infected Vero cell culture supernatant (prepared in parallel to ZIKV stocks) diluted in PBS to a final volume of 200 μL. LCMV Armstrong was injected i.p. at 2×10^5^ PFU in a final volume of 200 μL of PBS.

### Antibodies and Flow Cytometry Staining

The following antibodies were used in an appropriate combination of fluorochromes: CD3ε (clone 145-2C11, BioLegend), CD4 (clone GK1.5, BD Biosciences), CD8α (clone 53–6.7, BioLegend), CD11a (clone M17/4, BioLegend), CD11c (clone N418, eBioscience), CD19 (clone eBio1D3, eBiosciences), CD40 (clone 1C10, eBioscience), CD49d (clone R1-2, BioLegend), CD62L (clone MEL-14, BioLegend), CD69 (clone H1-2F3, eBioscience), CD80 (clone 16-10A1, eBioscience), CD86 (clone GL1, eBioscience), CD127 (clone A7R34, BioLegend), FoxP3 (clone FJK-16s, eBioscience), granzyme B (clone GB11, BioLegend), IFN-γ (clone XMG1.2, eBioscience), IL-2 (clone JES6-5H4, BioLegend), IL-5 (clone TRFK5, BioLegend), IL-17A (clone eBio 17B7, eBioscience), KLRG1 (clone 2F1/KLRG1, BioLegend), MHC-II (clone M5/114.15.2, eBioscience), NK1.1 (clone PK136, eBioscience), T-bet (clone 4B10, BioLegend), TCR Vα2 (clone B20.1, eBioscience), Thy1.1 (clone OX-7, BioLegend), Thy1.2 (clone 53–2.1, BioLegend), TNF-α (clone MP6-XT22, BioLegend) and appropriate isotype controls. Blood was collected and erythrocytes lysed using Vitalyse (Cedarlane, BioE), cells were incubated with TruStain fcX (anti-mouse CD16/CD32, clone 93, BioLegend) and stained using indicated antibodies, and samples were fixed using IC Fixation Buffer (eBioscience). Spleens were isolated on indicated dpi and mechanically disrupted to generate single-cell suspensions. Erythrocytes were lysed with ACK buffer, cells were incubated with TruStain fcX (anti-mouse CD16/CD32, clone 93, BioLegend) and stained with the indicated antibodies, and samples were fixed using IC Fixation Buffer (eBioscience). Intracellular staining for cytokines and granzyme B was performed using Perm/Wash Buffer (eBioscience). Intracellular staining for transcription factors was performed using the FoxP3 staining buffer set (eBioscience) according to the manufacturer’s instructions. Samples were analyzed with a BD LSRFortessa flow cytometer (BD Biosciences) and FlowJo software (Tree Star).

### Adoptive Transfers

Naïve T cell receptor transgenic (TCR-tg) CD4^+^ (10,000 SMARTA Thy1.1/1.2 cells; TCR-tg cells specific for LCMV epitope GP_61-80_) [27] or CD8^+^ (5,000 P14 Thy1.2/1.2 cells; TCR-tg cells specific for LCMV epitope GP_33-41_) T cells were adoptively transferred into naïve C57BL/6 (Thy1.1/1.1) recipient mice one day before infection [28]. The following day mice were infected with LCMV Armstrong strain (2×10^5^ PFU i.p.).

### *Ex Vivo* Stimulation

At 7 dpi spleens were isolated and mechanically disrupted to generate single-cell suspensions. Erythrocytes were lysed with ACK buffer, and stimulated for 3 h at 37°C/5% CO_2_ in 5 ng/mL PMA and 500 ng/mL ionomycin, 5 h with 1 μg/well plate-bound anti-CD3 antibody, or 5.5 h with 200 nM of individual peptides (ZIKV Env_294-302_, NS2A_1272-1280_, NS2A-NS2B_1350-1358_, NS4B_2329-2337_, NS4B_2371-2379_, or NS5_3066-3074_, numbered using the ZIKV MR766 reference strain polyprotein [Genbank accession: LC002520]; or LCMV GP_33-41_) in the presence of Brefeldin A. Type of stimulation used is indicated in the corresponding figure legend. Anti-CD3 antibody was bound to 96-well plates by incubating in 100 μL PBS for 2 h at 37°C/5% CO_2_.

### Quantification of Viral Burden

ZIKV-, UV-inactivated ZIKV or mock-infected mice were euthanized 6, 12, 24, 48 or 72 h post-infection and spleens were collected and weighed. Total RNA was harvested by Trizol extraction according to the manufacturer’s instructions and RNA concentration was determined using a NanoDrop 2000 spectrophotometer. Quantification of ZIKV RNA in the mouse spleen was determined by TaqMan one-step quantitative real time PCR (qRT-PCR) on a Bio-Rad CFX96 Touch Real-Time System using an iTaQ Universal Probe One-Step Kit (Bio-Rad) under standard cycling conditions. The primer set used to detect ZIKV RNA included the following primers: forward, 5’-CCG CTG CCC AAC ACA AG-3’; reverse, 5’-CCA CTA ACG TTC TTT TGC AGA CAT-3’; probe, 5’-/56-FAM/AGC CTA CCT/ZEN/TGA CAA GCA ATC AGA CAC TCA A/3IABkFQ/-3’ (Integrated DNA Technologies) [11, 41]. A standard curve was generated of C_t_ value versus log_10_ PFU using serial 10-fold dilutions of ZIKV RNA extracted from previously titrated viral stocks. Viral burden is expressed as PFU equivalents per gram of tissue after comparison with the standard curve (S3 Fig). The limit of detection was set as the average PFU equivalent per gram of tissue from the mock-infected samples.

### qRT-PCR Analysis

Total RNA was extracted as described above and reverse transcribed using the iScript Reverse Transcription Supermix for RT-qPCR (Bio-Rad) according to the manufacturer’s instructions. qRT-PCR was performed in duplicate in 96-well PCR plates (Bio-Rad) using SensiFAST SYBR Lo-ROX mix (Bioline) in a Bio-Rad CFX96 Touch Real-Time System under standard cycling conditions. Relative mRNA levels were calculated using the ΔΔCT method [42] using TATA-binding protein (TBP) expression as an internal control, and plotted as fold change by normalizing to mock-infected samples. The following primers were used to detect IFN-α, IFN-β and TBP mRNA: IFN-α Forward: 5’-TGT CTG ATG CAG CAG GTG G-3’; IFN-α Reverse: 5’-AAG ACA GGG CTC TCC AGA C-3’; IFN- β Forward: 5’-CCA TCC AAG AGA TGC TCC AG-3’; IFN- β Reverse: 5’-GTG GAG AGC AGT TGA GGA CA-3’; TBP Forward: 5’-TGG AAT TGT ACC GCA GCT TCA-3’; TBP Reverse: 5’-ACT GCA AAT CGC TTG GG-3’.

### Identification of Potential MHC-I Binding Peptides

The MHC-I binding predictions were made using the Immune Epitope Database (IEDB) analysis resource Consensus tool [36], which combines predictions from ANN aka NetMHC (4.0) [43–45], SMM [46] and Comblib [47]. The ZIKV polyprotein sequence used for analysis was the PLCal_ZV isolate (Genbank accession KF993678) [29]. The following 9mer peptides were identified and synthesized (BioBasic) for CD8^+^ T cell restimulation assays (numbered for their position in the ZIKV MR766 polyprotein, Genbank accession LC002520): Env_294-302_: IGVSNRDFV; NS2A_1272-1280_: MVLINGFAL; NS2A-NS2B_1350-1358_: TAVRLVDPI; NS4B_2329-2337_: TSYNNYSLM; NS4B_2371-2379_: SQLTPLTLI; NS5_3066-3074_: FDLENEALI.

### Statistical Analyses

Data were analyzed using GraphPad Prism7 software. Specific tests for determining statistical significance are indicated in the figure legends. P values of less than 0.05 were considered statistically significant.

### Ethics Statement

All animal procedures were carried out in accordance with the Canadian Council on Animal Care and were approved by the McGill University Animal Care Committee (Protocol #7800).

## Supporting Information

S1 Fig T cells upregulate surrogate marker expression following LCMV infection. **(A)** Experimental design. **(B)** Morbidity was analyzed on indicated days by measuring weights, presented as a percentage of day 0 weight. **(C)** Analysis of naïve recipient blood of CD11a^**+**^CD49d^**+**^ and CD8α^lo^CD11a^hi^ expression for CD4^**+**^ and CD8^**+**^ T cells, respectively, prior to adoptive transfer. **(D)** Analysis of CD11a^**+**^CD49d^**+**^ expression on naïve SMARTA CD4^**+**^ T cell receptor (TCR)-transgenic donor cells prior to transfer. **(E)** Analysis of CD8α^lo^CD11a^hi^ expression of naïve P14 CD8^**+**^ TCR-transgenic donor cells prior to transfer. **(F)** Analysis of day 8 blood of CD11a^**+**^CD49d^**+**^ expression for CD4^**+**^ T cells (endogenous and SMARTA TCR-transgenic) (top) and CD8α^lo^CD11a^hi^ expression for CD8^**+**^ T cells (endogenous and P14 TCR-transgenic) (bottom). **(G)** Analysis of day 8 spleen of CD11a^**+**^CD49d^**+**^ expression for CD4^**+**^ T cells (endogenous and SMARTA TCR-transgenic) (top) and CD8α^lo^CD11a^hi^ expression for CD8^**+**^ T cells (endogenous and P14 TCR-transgenic) (bottom). Data are representative dot plots for two independent experiments, n = 3 mice per experiment.
(PDF)

S2 Fig Standard curve of ZIKV RNA serial dilutions measured by qRT-PCR. ZIKV RNA was extracted from a previously titrated viral stock, serially diluted (10-fold), and analyzed by TaqMan one-step quantitative reverse transcriptase PCR (qRT-PCR). C_t_ values obtained from qRT-PCR were plotted against plaque forming units (PFU) on a log_10_ scale to generate a standard curve. Data are pooled from two independent experiments.
(PDF)

S3 Fig Dendritic cell gating strategy and early T cell activation. **(A-D)** Gating strategy to identify splenic dendritic cells. Dendritic cells were identified by gating on **(A)** live cells, **(B)** singlets, **(C)** CD3^-^CD19^-^NK1.1^-^ cells (dump gate) and **(D)** MHC-II^+^CD11c^+^ dendritic cells. Representative histogram **(E),** percentage **(F)** and number **(G)** of CD69^+^ NK1.1^-^CD3^+^ total T cells from mock- (shaded histogram), UV-inactivated ZIKV- (open histogram with dashed line) and ZIKV-infected (open histogram with solid line) mice 2 dpi. Number on histogram indicates percentage of CD69^+^ cells from ZIKV-infected sample. Error bars represent mean ± SEM. Data are pooled from two independent experiments, n = 3 mice per group per experiment. Data in **(F** and **G)** were analyzed by one-way ANOVA with Tukey’s post-test of multiple comparisons. ****p<0.0001.
(PDF)

S4 Fig Dose-dependence of T cell response induced by ZIKV infection. Representative histograms of CD11a^+^CD49d^+^ CD4^+^ T cells from mouse spleens 7 dpi i.v. with **(A)** mock-infected media or **(B)** 10^4^ PFU, **(C)** 10^5^ PFU or **(D)** 10^6^ PFU of ZIKV. Percentage **(E)** and number **(F)** of CD11a^+^CD49d^+^ CD4^+^ T cells from mice infected with mock-infected media or 10^4^ PFU, 10^5^ PFU or 10^6^ PFU of ZIKV. Representative histograms of CD8α^lo^CD11a^hi^ CD8^**+**^ T cells from mouse spleens 7 dpi i.v. with **(G)** mock-infected media or **(H)** 10^4^ PFU, **(I)** 10^5^ PFU or **(J)** 10^6^ PFU of ZIKV. Percentage **(K)** and number **(L)** of CD8α^lo^CD11a^hi^ CD8^**+**^ T cells from mice infected with mock-infected media or 10^4^ PFU, 10^5^ PFU or 10^6^ PFU of ZIKV. Error bars represent mean ± SEM. Data are pooled from two independent experiments, n = 3 mice per group per experiment. Data in (**E**, **F**, **K** and **L**) were analyzed by one-way ANOVA with Tukey’s post-test of multiple comparisons. *p<0.05; **p<0.005; ***p<0.0005; ****p<0.0001.
(PDF)

S5 Fig Antigen-experienced, but not naïve, T cells produce cytokines in response to CD3 stimulation. Representative plots of IFN-γ production by CD11a^-^CD49d^-^ (naïve) CD4^+^ T cells **(A)** and CD11a^+^CD49d^+^ (antigen-experienced) CD4^+^ T cells **(B)** in response to no stimulation (top) or plate-bound anti-CD3 stimulation (bottom) for 5 h at 37°C in the presence of Brefeldin A. **(C)** Percentage of IFN-γ^+^ naïve and antigen-experienced CD4^+^ T cells. Representative plots of IFN-γ production by CD8α^hi^CD11a^lo^ (naïve) CD8^**+**^ T cells **(D)** and CD8α^lo^CD11a^hi^ (antigen-experienced) CD8^+^ T cells **(E)** in response to no stimulation (top) or plate-bound anti-CD3 stimulation (bottom) for 5 h at 37°C in the presence of Brefeldin A. **(F)** Percentage of IFN-γ^+^ naïve and antigen-experienced CD4^+^ T cells. Error bars represent mean ± SEM. Data are pooled from two independent experiments, n = 3 mice per group per experiment.
(PDF)

S6 Fig Poly-functionality of CD11a^+^CD49d^+^ CD4^+^ T cells and CD8α^lo^CD11a^hi^ CD8^+^ T cells. Representative plots **(A-B)** and percentages **(C-D)** of IFN-γ^+^TNF-α^+^ CD11a^+^CD49d^+^ “double-producer” CD4^**+**^ T cells **(A)** and (**C)**, and IFN-γ^+^TNF-α^+^IL-2^+^ CD11a^+^CD49d^+^ “triple-producer” CD4^**+**^ T cells **(B)** and **(D).** Representative plot **(E)** and percentage **(F)** of IFN-γ^+^TNF-α^+^ CD8α^lo^CD11a^hi^ “double-producer” CD8^**+**^ T cells. Error bars represent mean ± SEM. Data are pooled from two independent experiments, n = 5 mice per experiment.
(PDF)

S7 Fig CD11a^+^CD49d^+^ CD4^+^ T cells do not produce Th2 or Th17 cytokines in response to ZIKV infection. Representative plots of IL-5 **(A)** and **(B),** and IL-17 **(C)** and **(D)** production by CD11a^+^CD49d^+^ CD4^**+**^ T cells in response to no stimulation **(A)** and **(C)** or PMA plus ionomycin stimulation **(B)** and **(D)**. **(E)** Percentage of IL-5 and IL-17 producing CD11a^+^CD49d^+^ CD4^**+**^ T cells. Representative plots of FoxP3 expression in CD4^**+**^ T cells from mock- **(F)** and ZIKV-infected **(G)** mice. Percentage **(H)** and number **(I)** of FoxP3^+^ CD4^**+**^ T cells from mock- and ZIKV-infected mice. Error bars represent mean ± SEM. Data are pooled from two independent experiments, n = 3 to 5 mice per group per experiment. Data in **(H)** and **(I)** were analyzed with a two-tailed, unpaired Student’s t test. ***p<0.0005.
(PDF)

S8 Fig LCMV infection induces robust CD4^+^ T cell activation and Th1 cytokine production by antigen-experienced CD4^+^ T cells. **(A)** Representative plot of CD11a^**+**^CD49d^**+**^ CD4^**+**^ T cells from the spleens of mice 7 dpi i.p. with 2×10^5^ PFU of LCMV. Percentage **(B)** and number **(C)** of CD11a^**+**^CD49d^**+**^ CD4^**+**^ T cells from LCMV-infected mice. Representative plots of IFN-γ **(D)**, TNF-α **(E)**, IL-2 **(F)**, IL-5 **(G)** and IL-17 **(H)** production by CD11a^**+**^CD49d^**+**^ CD4^**+**^ T cells in response to no stimulation (top) or stimulation with PMA and ionomycin for 3 h at 37°C (bottom) in the presence of Brefeldin A. Percentage **(I)** and number **(J)** of cytokine producing CD11a^**+**^CD49d^**+**^ CD4^**+**^ T cells. Error bars represent mean ± SEM. Data are pooled from two independent experiments, n = 3 mice per group per experiment.
(PDF)

S9 Fig LCMV infection induces robust CD8^+^ T cell activation and prototypical cytokine production by antigen-experienced CD8^+^ T cells. **(A)** Representative plot of CD8α^lo^CD11a^hi^ CD8^**+**^ T cells from the spleens of mice 7 dpi i.p. with 2×10^5^ PFU of LCMV. Percentage **(B)** and number **(C)** of CD8α^lo^CD11a^hi^ CD8^**+**^ T cells from LCMV-infected mice. Representative plots of IFN-γ **(D)**, TNF-α **(E)** and IL-2 **(F)** production by CD8α^lo^CD11a^hi^ CD8^**+**^ T cells in response to no stimulation (top) or stimulation with PMA and ionomycin for 3 h at 37°C (bottom) in the presence of Brefeldin A. Percentage **(G)** and number **(H)** of cytokine producing CD8α^lo^CD11a^hi^ CD8^**+**^ T cells. Error bars represent mean ± SEM. Data are pooled from two independent experiments, n = 3 mice per group per experiment.
(PDF)

S10 Fig Proportions of antigen-experienced expressing SLEC or MPEC phenotypes >100 dpi. **(A)** Representative plot of CD8α^lo^CD11a^hi^ CD8^**+**^ T cells from the peripheral blood of mice infected >100 days prior i.v. with 10^6^ PFU of ZIKV. Percentage **(B)** and number **(C)** of CD8α^lo^CD11a^hi^ CD8^**+**^ T cells from mice >100 dpi with ZIKV. **(D)** Representative plot of KLRG1 and CD127 expression on CD8α^lo^CD11a^hi^ CD8^**+**^ T cells from the peripheral blood of mice >100 dpi with ZIKV. Percentage **(E)** and number **(F)** of CD127^lo^KLRG1^hi^ SLECs and CD127^hi^KLRG1^lo^ MPECs >100 dpi with ZIKV. Error bars represent mean ± SEM. Data are pooled from two independent experiments, n = 3 or 4 mice per group per experiment.
(PDF)

S11 Fig IFN-γ production from mock- and UV-inactivated ZIKV-infected mice after peptide restimulation. **(A)** Representative plots of IFN-γ production from antigen-experienced CD8α^lo^CD11a^hi^ CD8^+^ T cells (top rows) and naïve CD8α^hi^CD11a^lo^ CD8^+^ T cells (bottom rows) from the spleens of mock-infected mice 7 dpi. Total splenocytes were incubated for 5.5 h at 37°C with media alone or 200 nM of the indicated peptide in the presence of Brefeldin A. **(B)** Representative plots of IFN-γ production from antigen-experienced CD8α^lo^CD11a^hi^ CD8^+^ T cells (top rows) and naïve CD8α^hi^CD11a^lo^ CD8^+^ T cells (bottom rows) from the spleens of mice 7 dpi with UV-inactivated ZIKV. Total splenocytes were incubated for 5.5 h at 37°C with media alone or 200 nM of the indicated peptide in the presence of Brefeldin A. Data are pooled from two independent experiments, n = 3 mice per group per experiment.
(PDF)

S12 Fig IFN-γ production from ZIKV- and LCMV-infected mice after ZIKV peptide restimulation from the NS2A, NS4B and NS5 proteins. **(A)** Representative plots of IFN-γ production from antigen-experienced CD8α^lo^CD11a^hi^ CD8^+^ T cells (top row) and naïve CD8α^hi^CD11a^lo^ CD8^+^ T cells (bottom row) from the spleens of ZIKV-infected mice 7 dpi. Total splenocytes were incubated for 5.5 h at 37°C with 200 nM of the indicated peptide in the presence of Brefeldin A. **(B)** Representative plots of IFN-γ production from antigen-experienced CD8α^lo^CD11a^hi^ CD8^+^ T cells (top row) and naïve CD8α^hi^CD11a^lo^ CD8^+^ T cells (bottom row) from the spleens of LCMV-infected mice 7 dpi. Total splenocytes were incubated for 5.5 h at 37°C with 200 nM of the indicated peptide in the presence of Brefeldin A. **(C-G)** Percentage of IFN-γ^+^ antigen-experienced CD8α^lo^CD11a^hi^ or naïve CD8α^hi^CD11a^lo^ CD8^+^ T cells from mock-, UV-inactivated ZIKV-, ZIKV- or LCMV-infected mice 7 dpi after restimulation with 200 nM of NS2A_1272-1280_ **(C)**, NS2A-NS2B_1350-1358_ **(D)**, NS4B_2329-2337_ **(E)**, NS4B_2371-2379_ **(F)** or NS5_3066-3074_ **(G)** for 5.5 h at 37°C in the presence of Brefeldin A. Error bars represent mean ± SEM. Data are pooled from two independent experiments, n = 3 mice per group per experiment. Data in (**C**, **D**, **E**, **F** and **G**) were analyzed with a two-tailed, paired Student’s t test. *p<0.05; **p<0.005.
(PDF)
