# Supplementary figures and images for: Analysis of the T Cell Response to Zika Virus and Identification of a Novel CD8+ T Cell Epitope in Immunocompetent Mice
Source: PLoS Pathog. 2017 Feb 23;13(2):e1006184. doi: 10.1371/journal.ppat.1006184 (PMC5322871; doi:10.1371/journal.ppat.1006184)

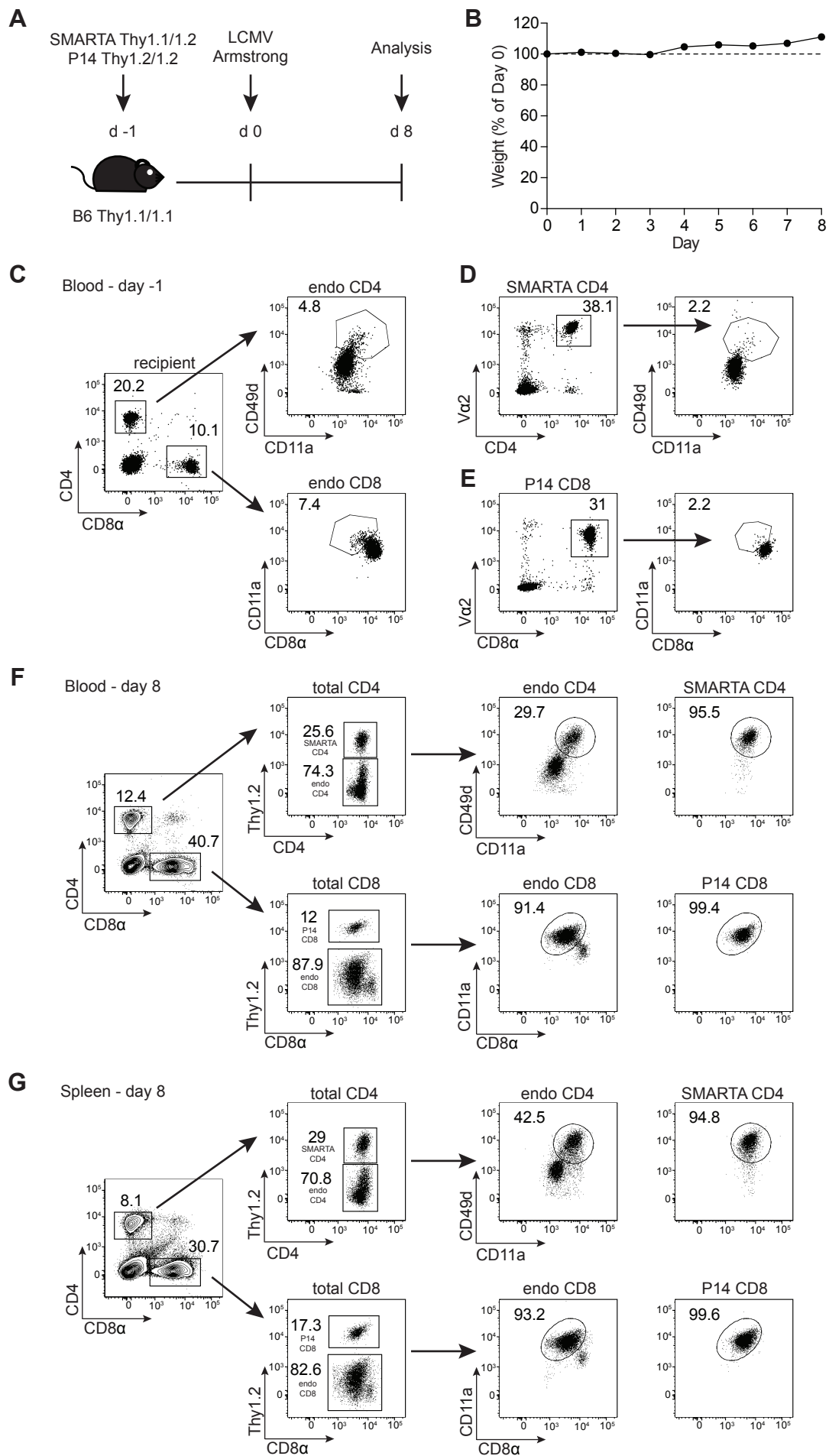

Supplement: S1 Fig — (A) Experimental design. (B) Morbidity was analyzed on indicated days by measuring weights, presented as a percentage of day 0 weight. (C) Analysis of naïve recipient blood of CD11a+CD49d+ and CD8αloCD11ahi expression for CD4+ and CD8+ T cells, respectively, prior to adoptive transfer. (D) Analysis of CD11a+CD49d+ expression on naïve SMARTA CD4+ T cell receptor (TCR)-transgenic donor cells prior to transfer. (E) Analysis of CD8αloCD11ahi expression of naïve P14 CD8+ TCR-transgenic donor cells prior to transfer. (F) Analysis of day 8 blood of CD11a+CD49d+ expression for CD4+ T cells (endogenous and SMARTA TCR-transgenic) (top) and CD8αloCD11ahi expression for CD8+ T cells (endogenous and P14 TCR-transgenic) (bottom). (G) Analysis of day 8 spleen of CD11a+CD49d+ expression for CD4+ T cells (endogenous and SMARTA TCR-transgenic) (top) and CD8αloCD11ahi expression for CD8+ T cells (endogenous and P14 TCR-transgenic) (bottom). Data are representative dot plots for two independent experiments, n = 3 mice per experiment. (PDF) [file ppat.1006184.s001.pdf]

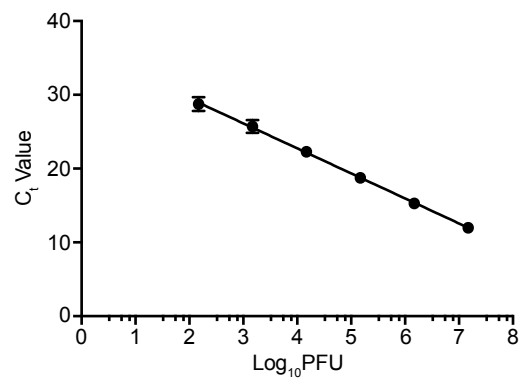

Supplement: S2 Fig — ZIKV RNA was extracted from a previously titrated viral stock, serially diluted (10-fold), and analyzed by TaqMan one-step quantitative reverse transcriptase PCR (qRT-PCR). Ct values obtained from qRT-PCR were plotted against plaque forming units (PFU) on a log10 scale to generate a standard curve. Data are pooled from two independent experiments. (PDF) [file ppat.1006184.s002.pdf]

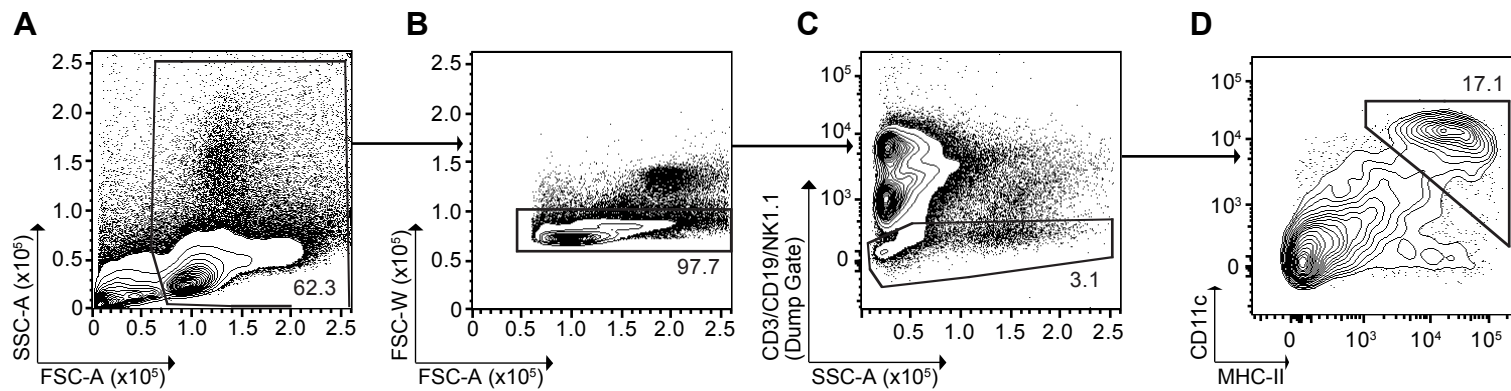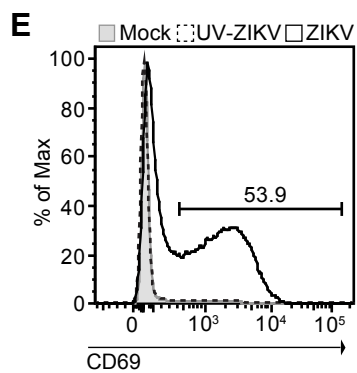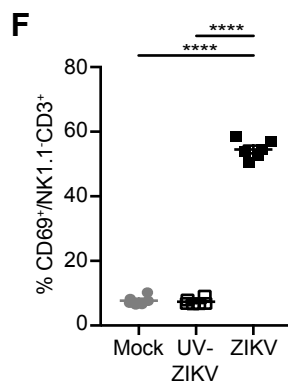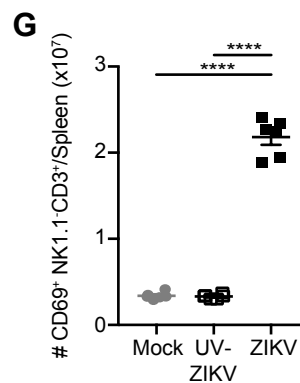

Supplement: S3 Fig — (A-D) Gating strategy to identify splenic dendritic cells. Dendritic cells were identified by gating on (A) live cells, (B) singlets, (C) CD3-CD19-NK1.1- cells (dump gate) and (D) MHC-II+CD11c+ dendritic cells. Representative histogram (E), percentage (F) and number (G) of CD69+ NK1.1-CD3+ total T cells from mock- (shaded histogram), UV-inactivated ZIKV- (open histogram with dashed line) and ZIKV-infected (open histogram with solid line) mice 2 dpi. Number on histogram indicates percentage of CD69+ cells from ZIKV-infected sample. Error bars represent mean ± SEM. Data are pooled from two independent experiments, n = 3 mice per group per experiment. Data in (F and G) were analyzed by one-way ANOVA with Tukey’s post-test of multiple comparisons. ****p<0.0001. (PDF) [file ppat.1006184.s003.pdf]

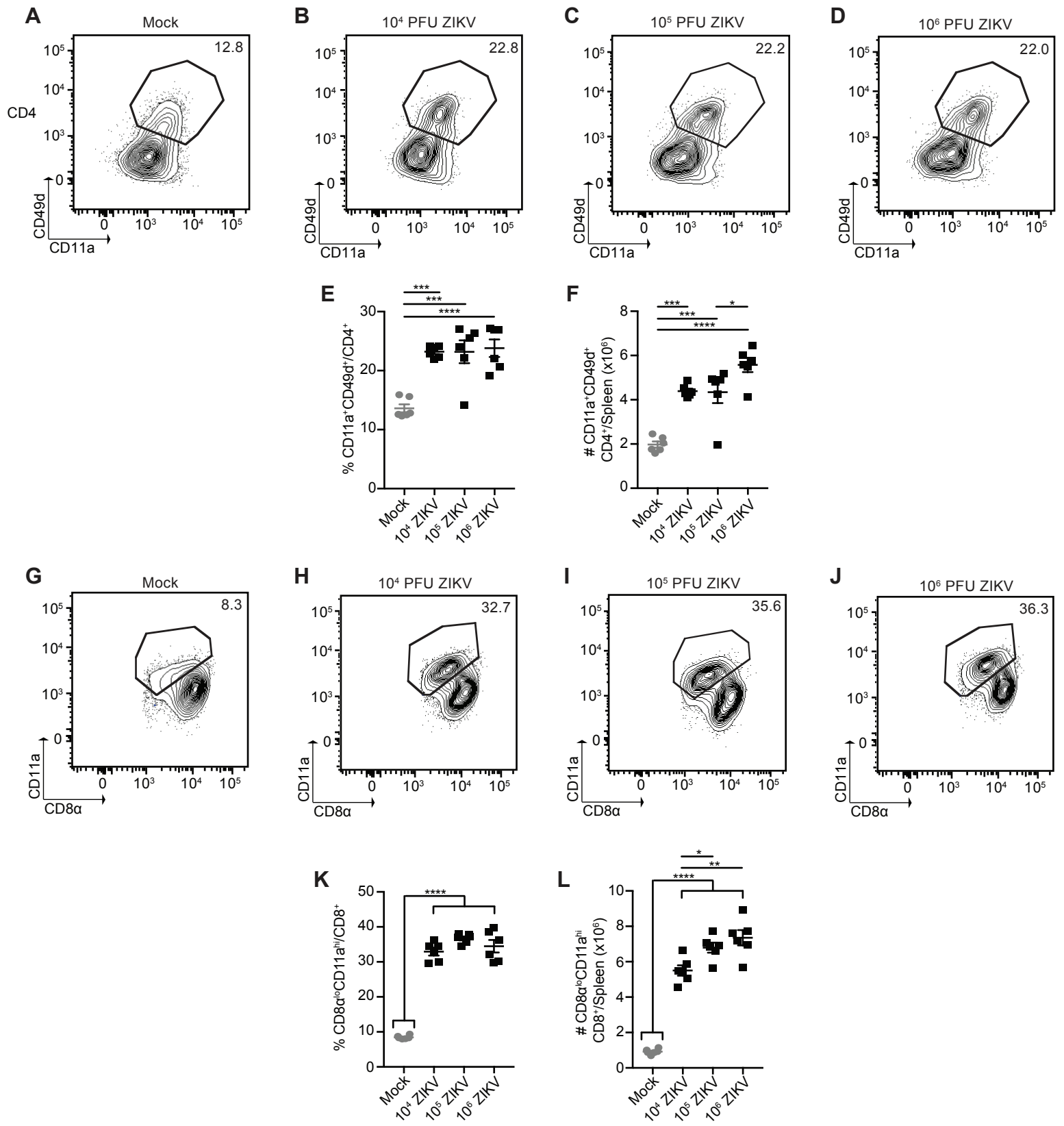

Supplement: S4 Fig — Representative histograms of CD11a+CD49d+ CD4+ T cells from mouse spleens 7 dpi i.v. with (A) mock-infected media or (B) 104 PFU, (C) 105 PFU or (D) 106 PFU of ZIKV. Percentage (E) and number (F) of CD11a+CD49d+ CD4+ T cells from mice infected with mock-infected media or 104 PFU, 105 PFU or 106 PFU of ZIKV. Representative histograms of CD8αloCD11ahi CD8+ T cells from mouse spleens 7 dpi i.v. with (G) mock-infected media or (H) 104 PFU, (I) 105 PFU or (J) 106 PFU of ZIKV. Percentage (K) and number (L) of CD8αloCD11ahi CD8+ T cells from mice infected with mock-infected media or 104 PFU, 105 PFU or 106 PFU of ZIKV. Error bars represent mean ± SEM. Data are pooled from two independent experiments, n = 3 mice per group per experiment. Data in (E, F, K and L) were analyzed by one-way ANOVA with Tukey’s post-test of multiple comparisons. *p<0.05; **p<0.005; ***p<0.0005; ****p<0.0001. (PDF) [file ppat.1006184.s004.pdf]

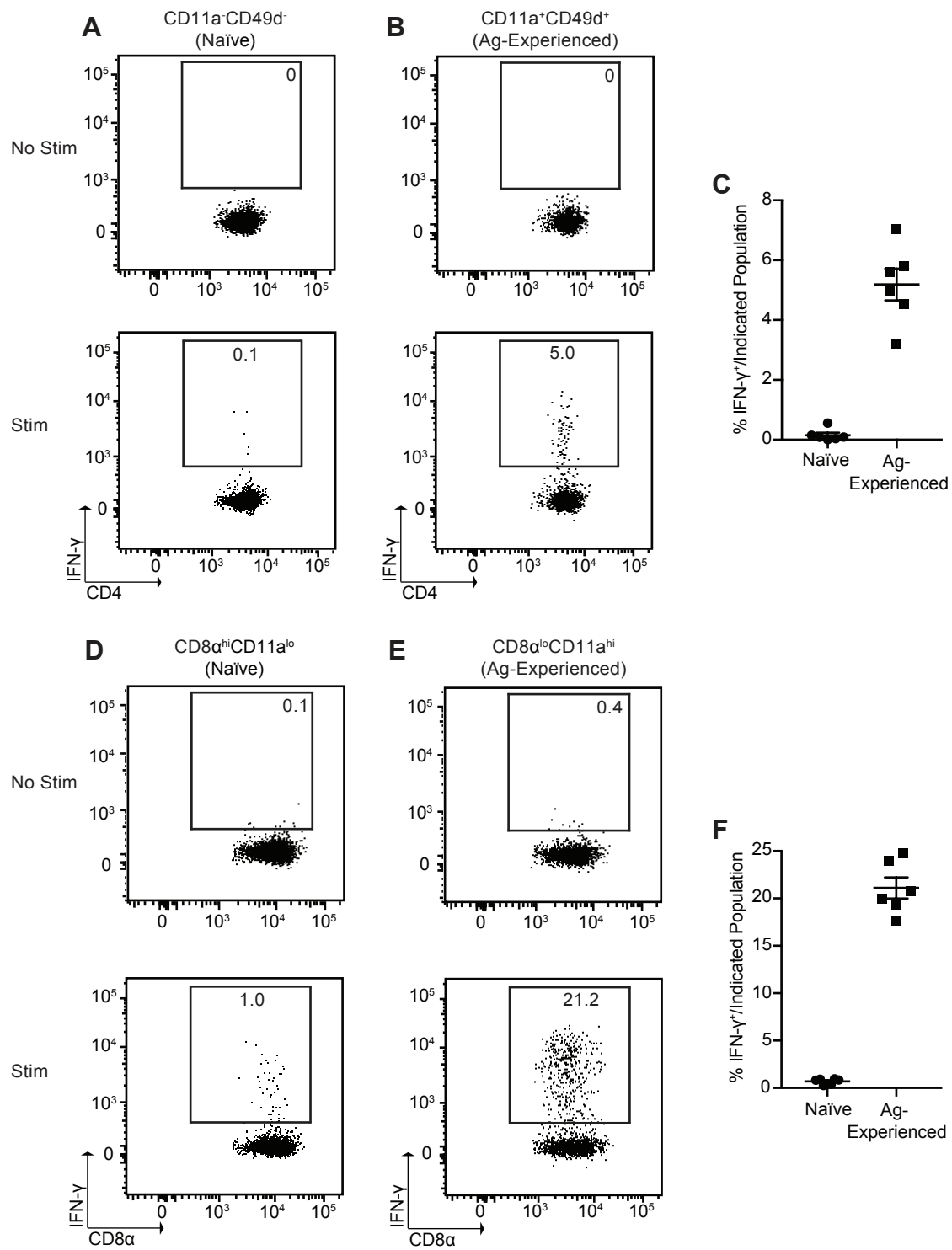

Supplement: S5 Fig — Representative plots of IFN-γ production by CD11a-CD49d- (naïve) CD4+ T cells (A) and CD11a+CD49d+ (antigen-experienced) CD4+ T cells (B) in response to no stimulation (top) or plate-bound anti-CD3 stimulation (bottom) for 5 h at 37°C in the presence of Brefeldin A. (C) Percentage of IFN-γ+ naïve and antigen-experienced CD4+ T cells. Representative plots of IFN-γ production by CD8αhiCD11alo (naïve) CD8+ T cells (D) and CD8αloCD11ahi (antigen-experienced) CD8+ T cells (E) in response to no stimulation (top) or plate-bound anti-CD3 stimulation (bottom) for 5 h at 37°C in the presence of Brefeldin A. (F) Percentage of IFN-γ+ naïve and antigen-experienced CD4+ T cells. Error bars represent mean ± SEM. Data are pooled from two independent experiments, n = 3 mice per group per experiment. (PDF) [file ppat.1006184.s005.pdf]

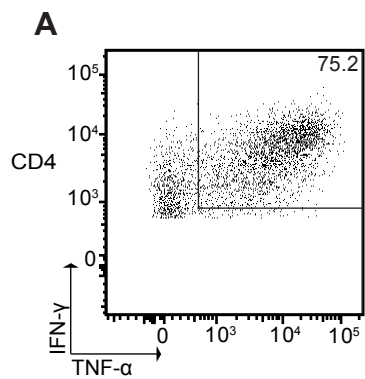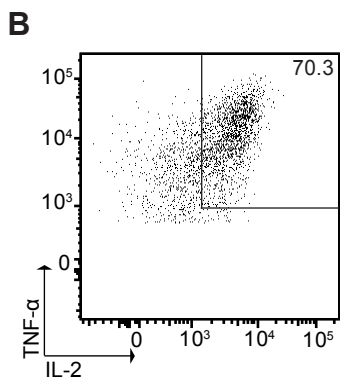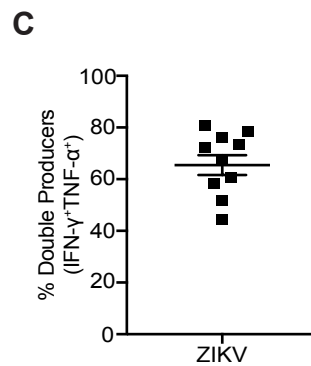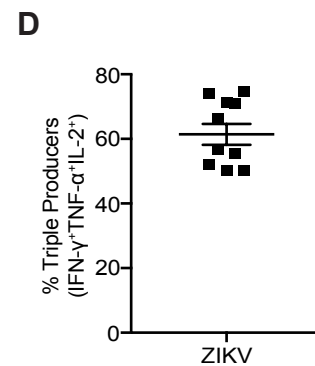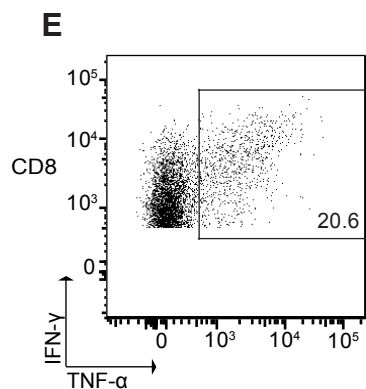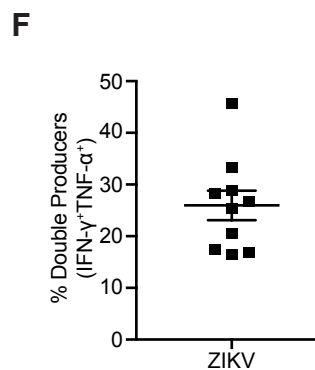

Supplement: S6 Fig — Representative plots (A-B) and percentages (C-D) of IFN-γ+TNF-α+ CD11a+CD49d+ “double-producer” CD4+ T cells (A) and (C), and IFN-γ+TNF-α+IL-2+ CD11a+CD49d+ “triple-producer” CD4+ T cells (B) and (D). Representative plot (E) and percentage (F) of IFN-γ+TNF-α+ CD8αloCD11ahi “double-producer” CD8+ T cells. Error bars represent mean ± SEM. Data are pooled from two independent experiments, n = 5 mice per experiment. (PDF) [file ppat.1006184.s006.pdf]

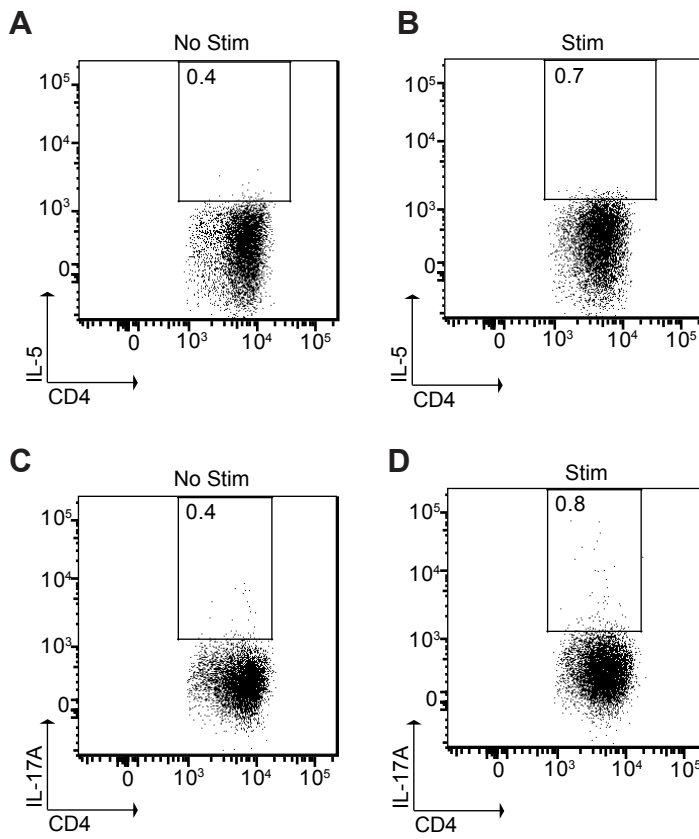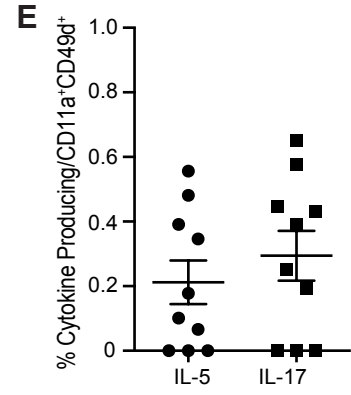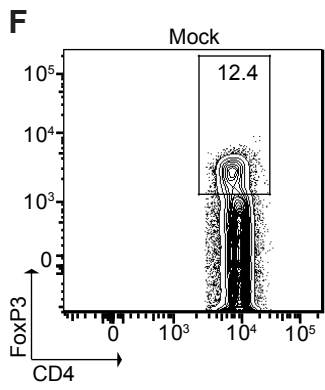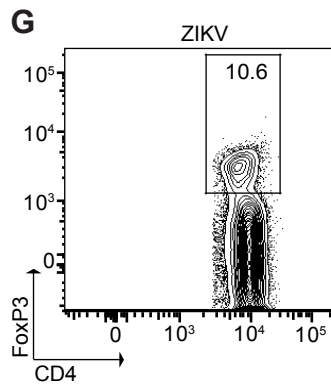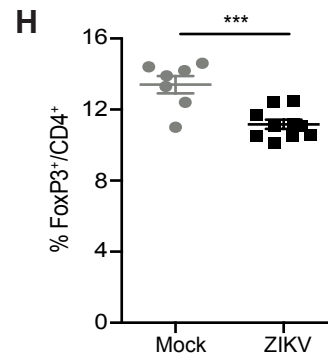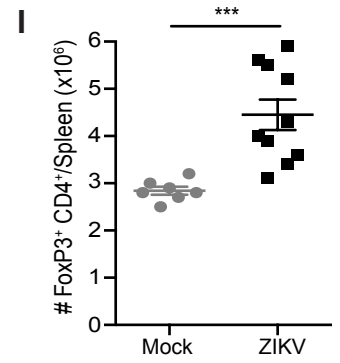

Supplement: S7 Fig — Representative plots of IL-5 (A) and (B), and IL-17 (C) and (D) production by CD11a+CD49d+ CD4+ T cells in response to no stimulation (A) and (C) or PMA plus ionomycin stimulation (B) and (D). (E) Percentage of IL-5 and IL-17 producing CD11a+CD49d+ CD4+ T cells. Representative plots of FoxP3 expression in CD4+ T cells from mock- (F) and ZIKV-infected (G) mice. Percentage (H) and number (I) of FoxP3+ CD4+ T cells from mock- and ZIKV-infected mice. Error bars represent mean ± SEM. Data are pooled from two independent experiments, n = 3 to 5 mice per group per experiment. Data in (H) and (I) were analyzed with a two-tailed, unpaired Student’s t test. ***p<0.0005. (PDF) [file ppat.1006184.s007.pdf]

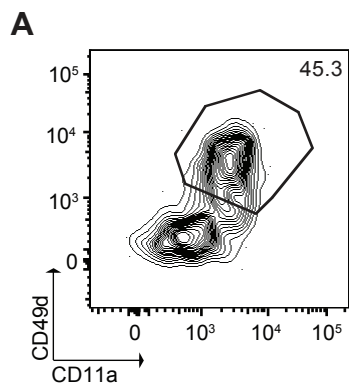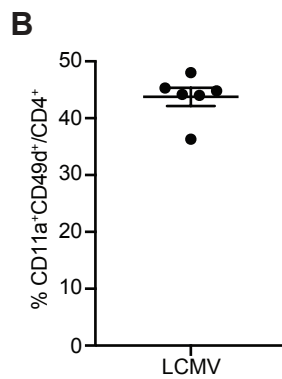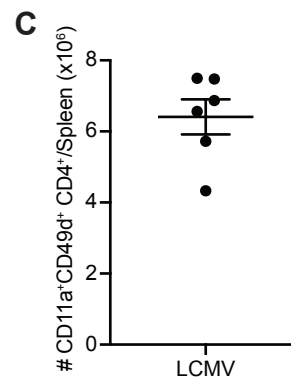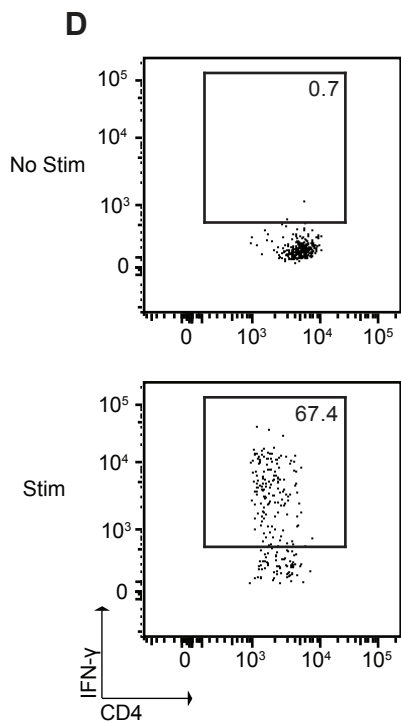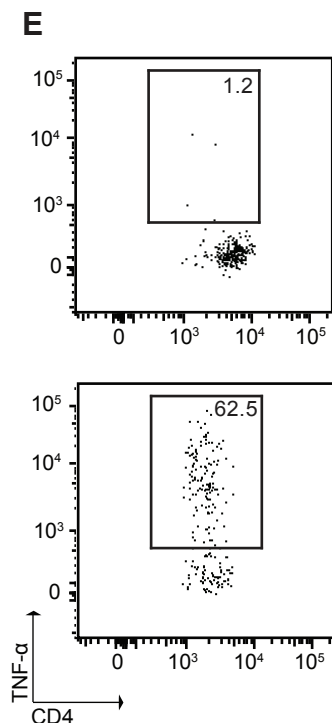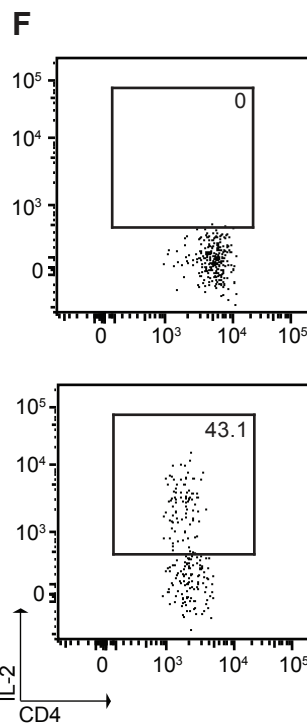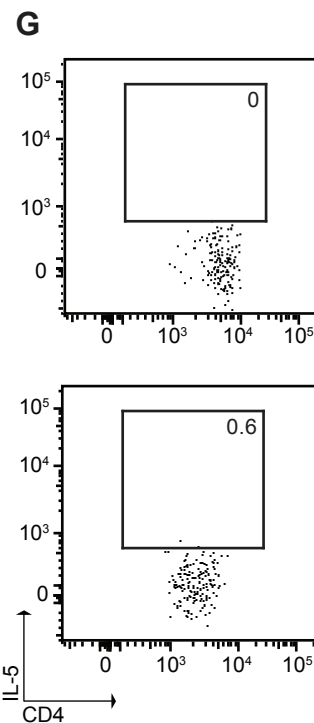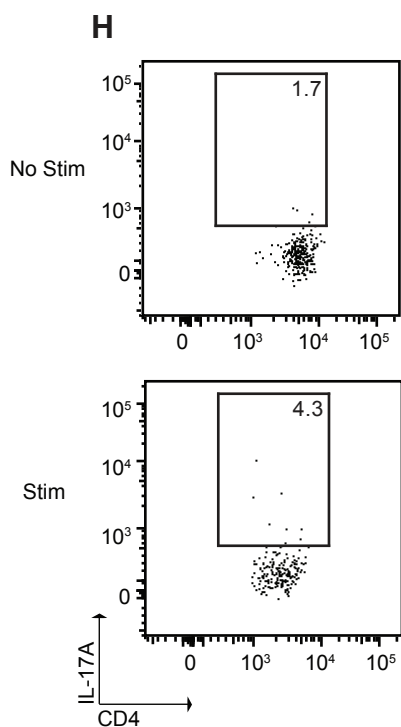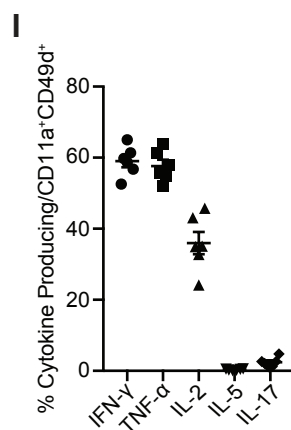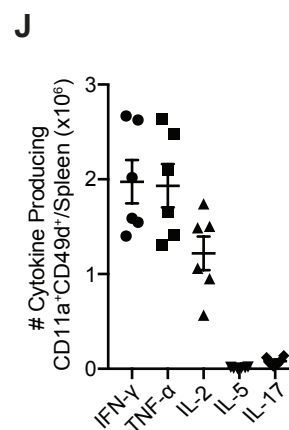

Supplement: S8 Fig — (A) Representative plot of CD11a+CD49d+ CD4+ T cells from the spleens of mice 7 dpi i.p. with 2×10^5^ PFU of LCMV. Percentage (B) and number (C) of CD11a+CD49d+ CD4+ T cells from LCMV-infected mice. Representative plots of IFN-γ (D), TNF-α (E), IL-2 (F), IL-5 (G) and IL-17 (H) production by CD11a+CD49d+ CD4+ T cells in response to no stimulation (top) or stimulation with PMA and ionomycin for 3 h at 37°C (bottom) in the presence of Brefeldin A. Percentage (I) and number (J) of cytokine producing CD11a+CD49d+ CD4+ T cells. Error bars represent mean ± SEM. Data are pooled from two independent experiments, n = 3 mice per group per experiment. (PDF) [file ppat.1006184.s008.pdf]

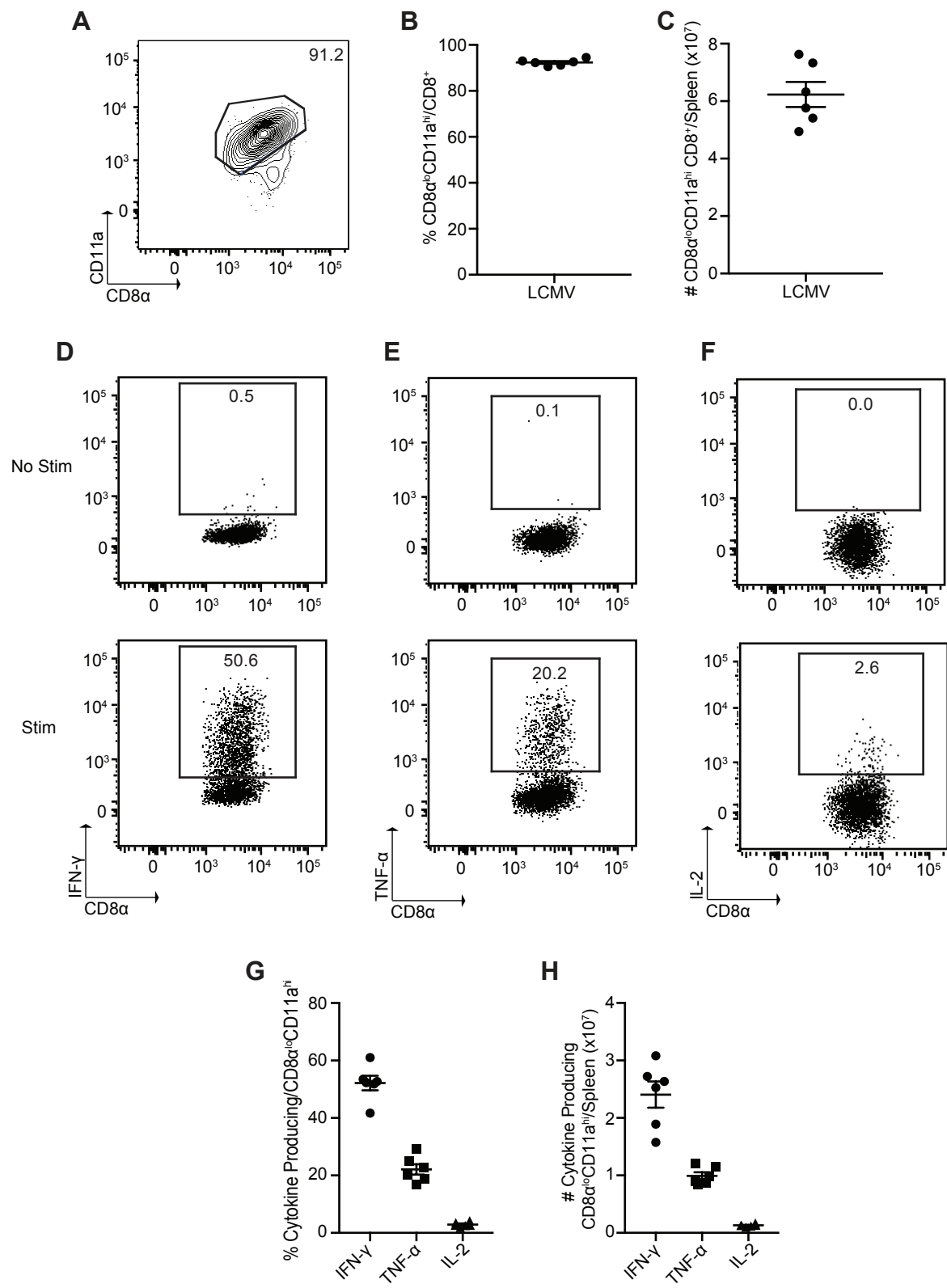

Supplement: S9 Fig — (A) Representative plot of CD8αloCD11ahi CD8+ T cells from the spleens of mice 7 dpi i.p. with 2×10^5^ PFU of LCMV. Percentage (B) and number (C) of CD8αloCD11ahi CD8+ T cells from LCMV-infected mice. Representative plots of IFN-γ (D), TNF-α (E) and IL-2 (F) production by CD8αloCD11ahi CD8+ T cells in response to no stimulation (top) or stimulation with PMA and ionomycin for 3 h at 37°C (bottom) in the presence of Brefeldin A. Percentage (G) and number (H) of cytokine producing CD8αloCD11ahi CD8+ T cells. Error bars represent mean ± SEM. Data are pooled from two independent experiments, n = 3 mice per group per experiment. (PDF) [file ppat.1006184.s009.pdf]

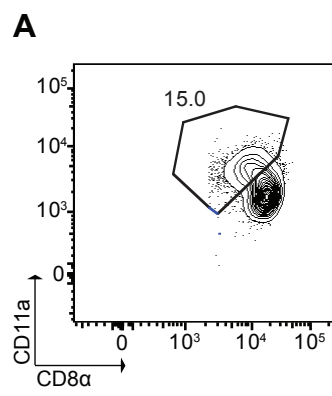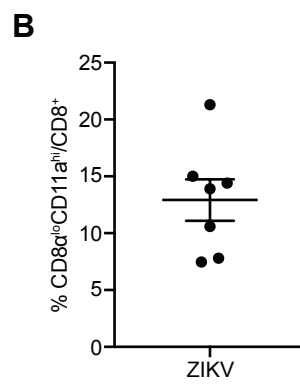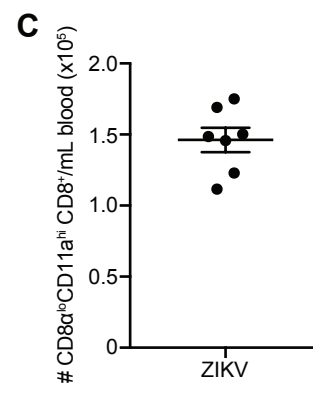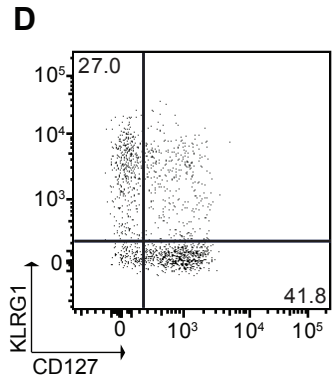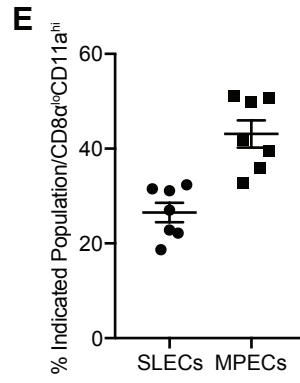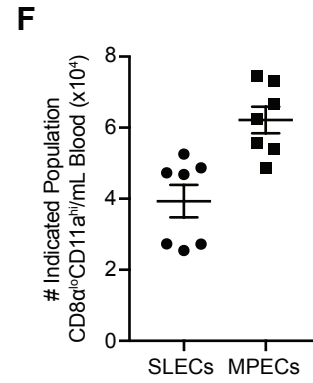

Supplement: S10 Fig — (A) Representative plot of CD8αloCD11ahi CD8+ T cells from the peripheral blood of mice infected >100 days prior i.v. with 106 PFU of ZIKV. Percentage (B) and number (C) of CD8αloCD11ahi CD8+ T cells from mice >100 dpi with ZIKV. (D) Representative plot of KLRG1 and CD127 expression on CD8αloCD11ahi CD8+ T cells from the peripheral blood of mice >100 dpi with ZIKV. Percentage (E) and number (F) of CD127loKLRG1hi SLECs and CD127hiKLRG1lo MPECs >100 dpi with ZIKV. Error bars represent mean ± SEM. Data are pooled from two independent experiments, n = 3 or 4 mice per group per experiment. (PDF) [file ppat.1006184.s010.pdf]

**A**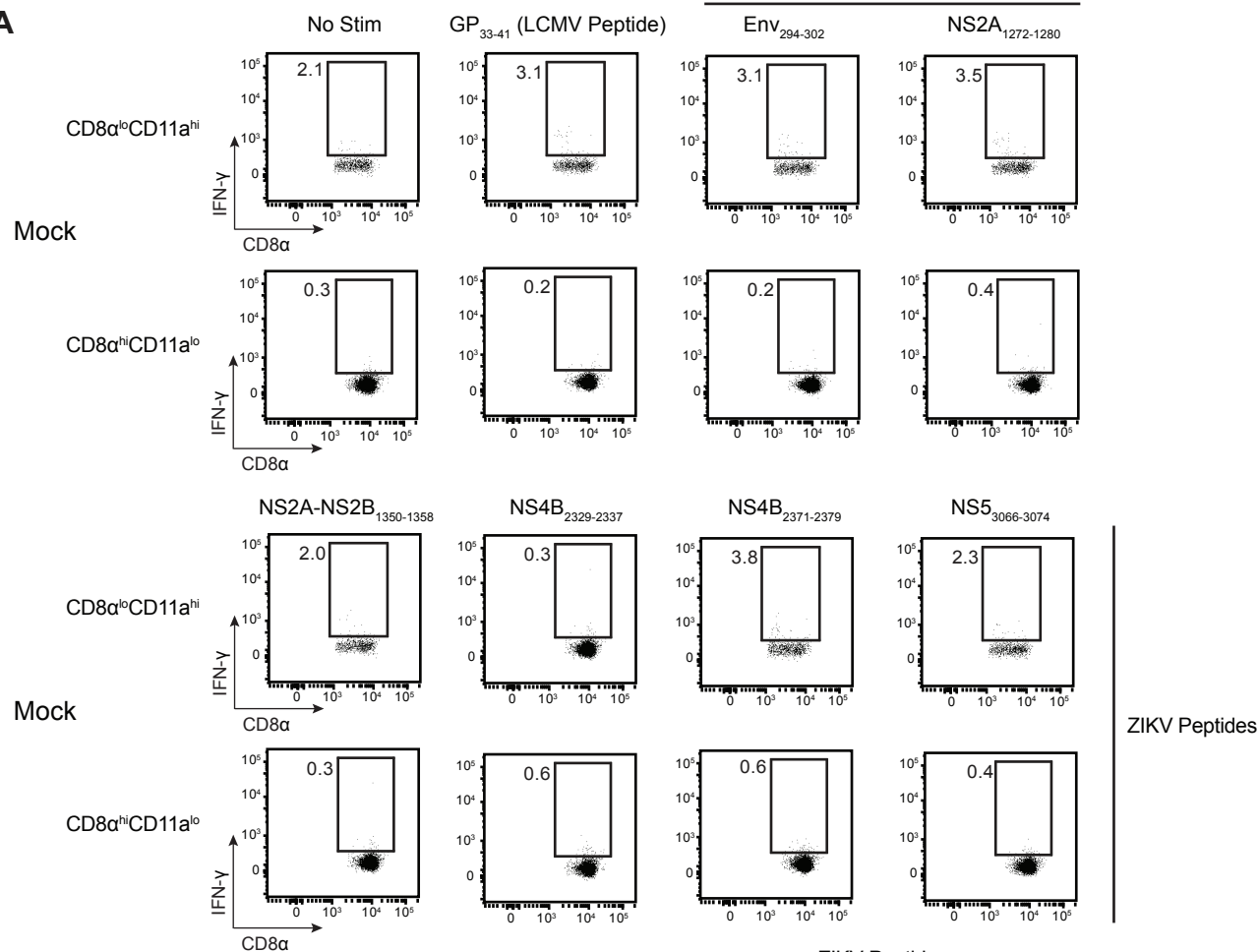**B**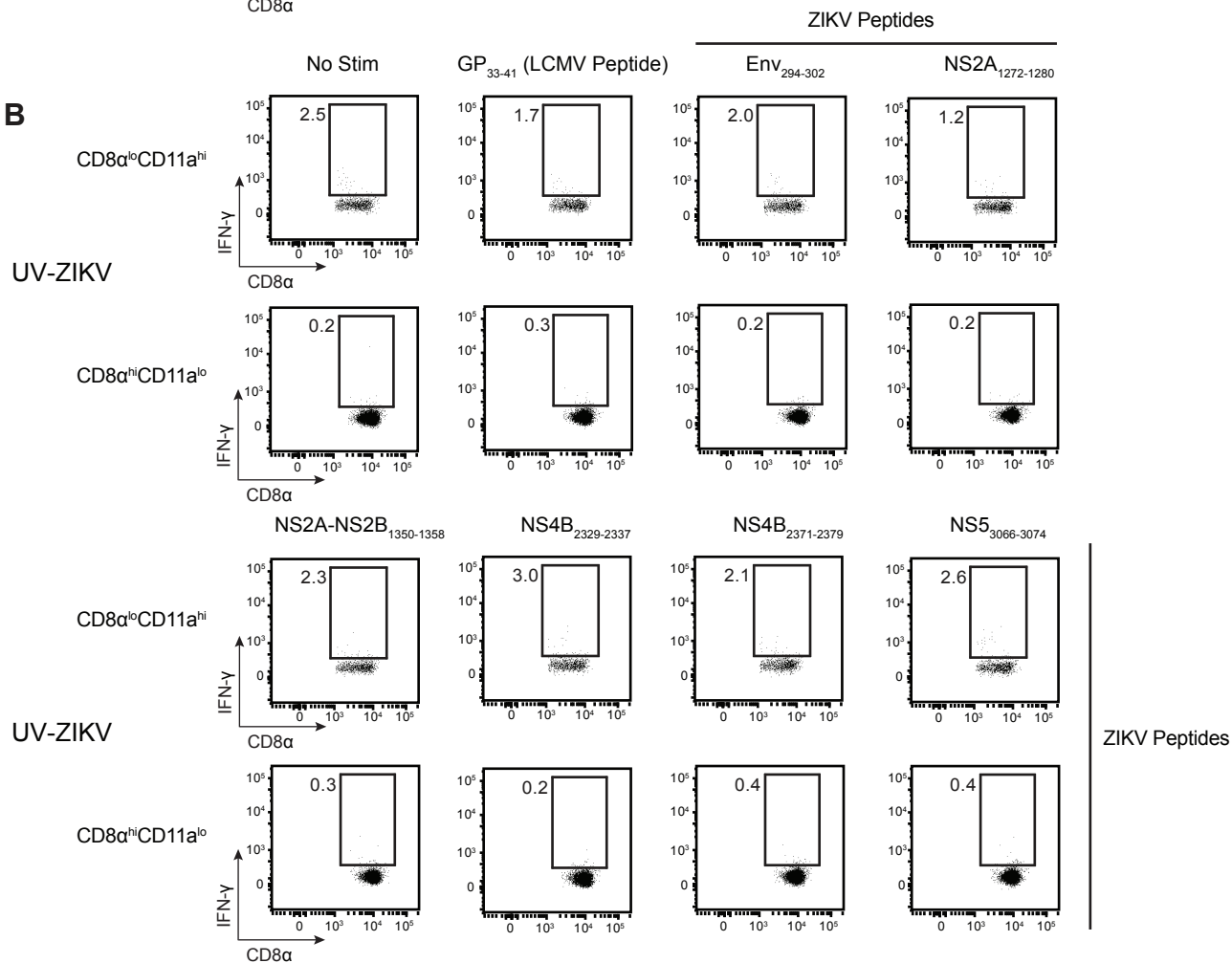

Supplement: S11 Fig — (A) Representative plots of IFN-γ production from antigen-experienced CD8αloCD11ahi CD8+ T cells (top rows) and naïve CD8αhiCD11alo CD8+ T cells (bottom rows) from the spleens of mock-infected mice 7 dpi. Total splenocytes were incubated for 5.5 h at 37°C with media alone or 200 nM of the indicated peptide in the presence of Brefeldin A. (B) Representative plots of IFN-γ production from antigen-experienced CD8αloCD11ahi CD8+ T cells (top rows) and naïve CD8αhiCD11alo CD8+ T cells (bottom rows) from the spleens of mice 7 dpi with UV-inactivated ZIKV. Total splenocytes were incubated for 5.5 h at 37°C with media alone or 200 nM of the indicated peptide in the presence of Brefeldin A. Data are pooled from two independent experiments, n = 3 mice per group per experiment. (PDF) [file ppat.1006184.s011.pdf]

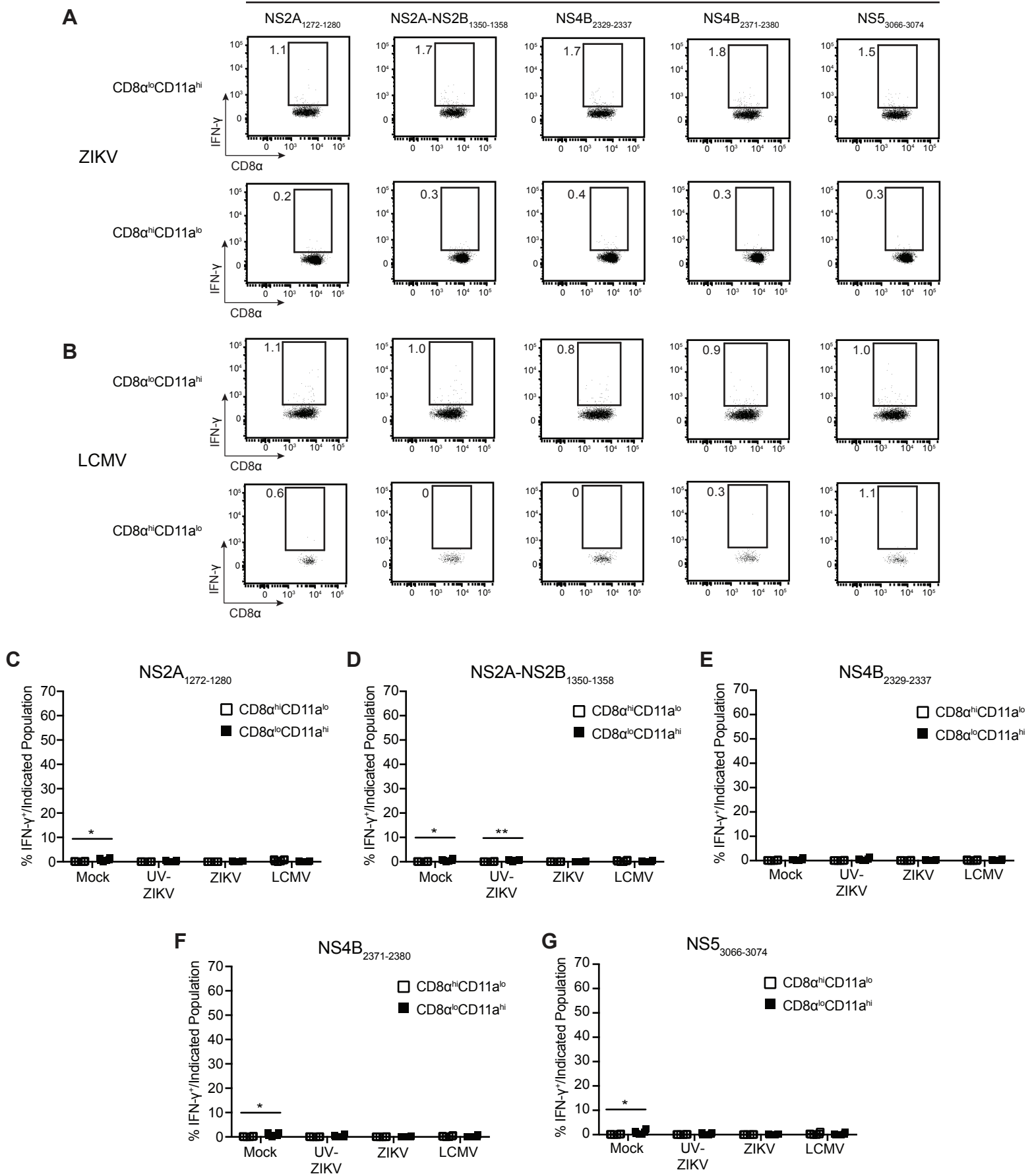

Supplement: S12 Fig — (A) Representative plots of IFN-γ production from antigen-experienced CD8αloCD11ahi CD8+ T cells (top row) and naïve CD8αhiCD11alo CD8+ T cells (bottom row) from the spleens of ZIKV-infected mice 7 dpi. Total splenocytes were incubated for 5.5 h at 37°C with 200 nM of the indicated peptide in the presence of Brefeldin A. (B) Representative plots of IFN-γ production from antigen-experienced CD8αloCD11ahi CD8+ T cells (top row) and naïve CD8αhiCD11alo CD8+ T cells (bottom row) from the spleens of LCMV-infected mice 7 dpi. Total splenocytes were incubated for 5.5 h at 37°C with 200 nM of the indicated peptide in the presence of Brefeldin A. (C-G) Percentage of IFN-γ+ antigen-experienced CD8αloCD11ahi or naïve CD8αhiCD11alo CD8+ T cells from mock-, UV-inactivated ZIKV-, ZIKV- or LCMV-infected mice 7 dpi after restimulation with 200 nM of NS2A1272-1280 (C), NS2A-NS2B1350-1358 (D), NS4B2329-2337 (E), NS4B2371-2379 (F) or NS53066-3074 (G) for 5.5 h at 37°C in the presence of Brefeldin A. Error bars represent mean ± SEM. Data are pooled from two independent experiments, n = 3 mice per group per experiment. Data in (C, D, E, F and G) were analyzed with a two-tailed, paired Student’s t test. *p<0.05; **p<0.005. (PDF) [file ppat.1006184.s012.pdf]
